# Learning from drops: AI-guided integration of liquid biopsy features in cancer studies

**DOI:** 10.3389/frai.2026.1937336

**Published:** 2026-10-23

**Authors:** Mireia Andueza, Pablo Villoslada-Blanco, Brune De Dreuille, Lola Alonso, Sergio Sabroso-Lasa, Klaus Pantel, Catherine Alix-Panabières, Evangelina López De Maturana, Núria Malats

**Affiliations:** 1 Genetic and Molecular Epidemiology Group (GMEG), Spanish National Cancer Research Centre (CNIO), Madrid, Spain; 2 CIBERONC, Madrid, Spain; 3 Department of Tumor Biology, University Medical Center Hamburg-Eppendorf, Hamburg, Germany; 4 European Liquid Biopsy Society, Hamburg, Germany; 5 Laboratory of Rare Human Circulating Cells (LCCRH) and Liquid Biopsy, University Medical Centre of Montpellier (CHUM), Montpellier, France; 6 CREEC (CREES), Unité Mixte de Recherches, IRD 224-CNRS 5290-Université de Montpellier, Montpellier, France

**Keywords:** Artificial Intelligence, cancer liquid biopsy, clinical utility, data integration, data preprocessing, feature selection, model interpretability, multimodal data fusion

## Abstract

Cancer is a major global health issue with rising incidence and mortality. Early detection, tumor characterization, and disease surveillance are crucial for timely, effective treatment and ultimately reduce mortality. Liquid biopsy (LB) has emerged as a valuable detection tool, offering a non-invasive method to detect tumor-derived biomarkers in body fluids and demonstrating translational potential. To increase biomarker sensitivity, high-throughput sequencing platforms deliver massive volumes of data. Artificial Intelligence (AI) is pivotal to enabling large-scale, complex data integration. This contribution assesses the current state of integrative AI-based research in the LB field and provides methodological guidance. First, we conducted a PubMed search and found that the literature is sparse in studies integrating LB features, particularly by applying AI. When adopting this approach, defining study objectives is crucial to guide subsequent methodological decisions, including study design, patient selection criteria, sample size, the nature of the LB features, and the metadata to collect. Specifically, we propose strategies and tools for data preprocessing, including normalization and batch-effect correction, as well as for handling outliers and missing data. Furthermore, we recommend various Machine/Deep Learning approaches for feature selection techniques to ensure model robustness, and we highlight the importance of undergoing rigorous internal and external validation of the selected models. Assessing clinical utility and interpretability is often overlooked but fundamental for real-world implementation. In conclusion, we provide the LB scientific community with AI-based methodological guidance to bridge the two fields and enhance the integrative analysis of LB features.

## Graphical abstract

**Figure:**
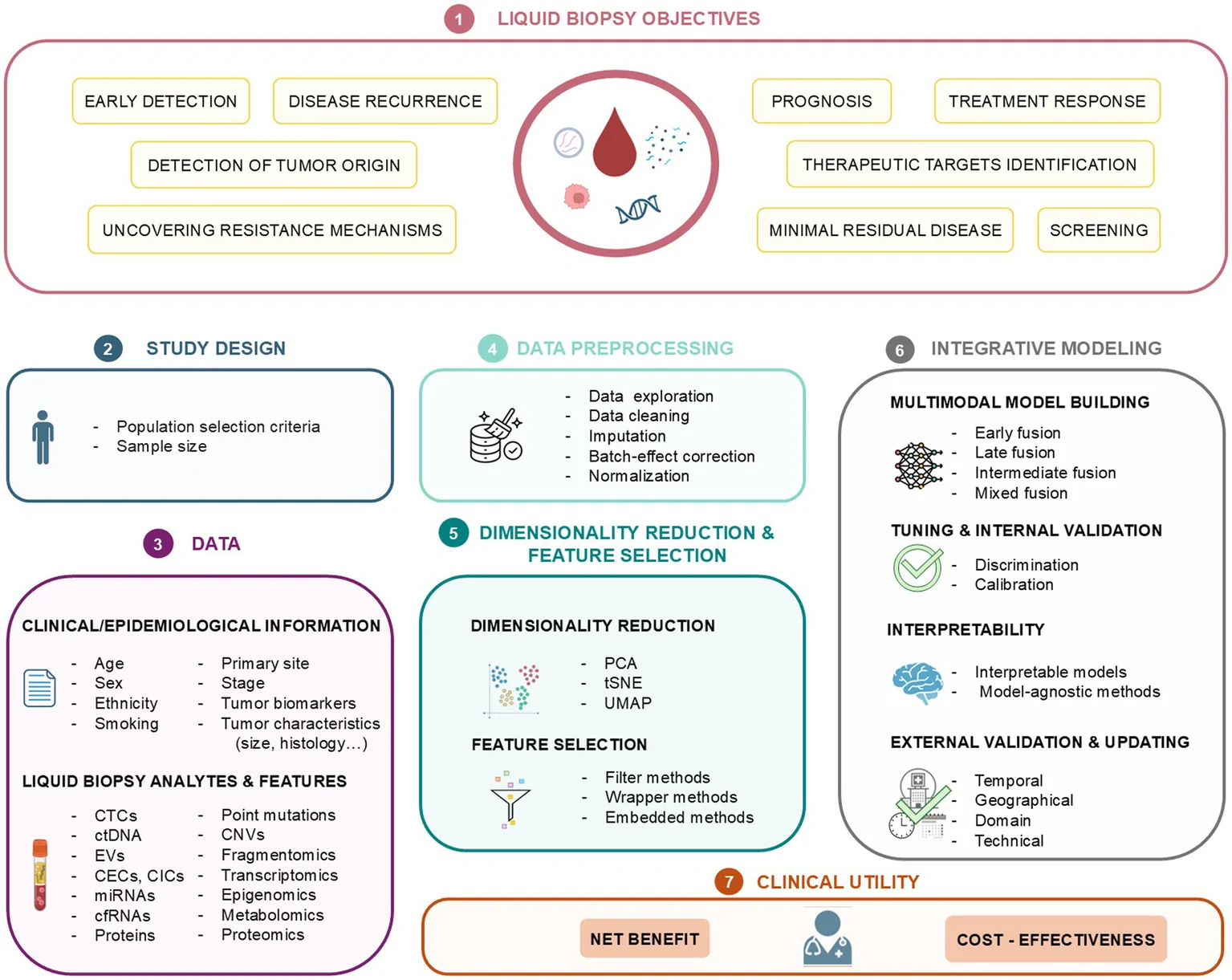
Methodological workflow for AI-based multiomics integration in liquid biopsy studies. The workflow summarizes the main steps discussed in this methodological guidance, from definition of the clinical objective and study design to data characterization, preprocessing, dimensionality reduction and feature selection, multimodal model building, internal and external validation, model interpretability and evaluation of clinical utility. CTCs, circulating tumor cells; ctDNA, circulating tumor DNA; TEPs, tumor-educated platelets; miRNA, microRNA; cfRNAs, cell-free RNAs.

## Introduction

Cancer is a major health issue worldwide, being the second leading cause of death and showing a rapid increase in incidence and mortality (Sung et al., 2021). According to estimates from the World Health Organization in 2022, nearly 10 million people died from cancer that year, and there were an estimated 20 million new cancer cases (World Health Organization, 2017a). This number is predicted to reach 27 million cases by 2040 (Wild et al., 2020).

Early detection and prediction of tumor response through tumor profiling are crucial for cancer control, enabling both effective patient treatment and reduced mortality rates (Loud and Murphy, 2017; World Health Organization, 2017b). Indeed, in solid cancers, tumors are known to present a large heterogeneity, and various subtypes can be found within a cancer type. Moreover, tumors can evolve temporally and spatially as metastases develop, and they acquire new genomic alterations and epigenetic changes during progression (Gerlinger et al., 2012; Dumbrava and Meric-Bernstam, 2018). Thus, molecular profiling is essential for diagnosing, treating, and monitoring cancer patients in a personalized manner.

The traditional methods for characterizing the disease include imaging and tissue biopsies, the latter of which are used for both morphological and molecular profiling of the tumor. However, tissue biopsy is invasive, and a single tumor biopsy may yield incomplete information due to intratumor heterogeneity, leading to underassessment of the tumor’s genomic landscape (Gerlinger et al., 2012). Given these limitations and the growing interest in personalized medicine, researchers have studied new biomarker-based techniques to monitor tumor evolution.

In particular, the concept of liquid biopsy (LB) was introduced in 2010 as a new diagnostic technique (Pantel and Alix-Panabières, 2010) and has generated considerable interest in recent years. This concept refers to the analysis of various biological fluids (blood, urine, saliva, and others), which contain circulating molecules and particles that can be shed by both primary tumors and metastases, such as circulating tumor cells (CTCs), cell-free DNA (cfDNA), circulating tumor DNA (ctDNA), tumor-derived extracellular vesicles (EVs), tumor-educated platelets (TEPs), and microRNAs (miRNA), among others (Alix-Panabières and Pantel, 2021; Lone et al., 2022). Several studies have shown the potential of LB feature analysis for early detection, tissue of origin (TOO) detection, minimal residual disease (MRD) monitoring, prognosis prediction, therapeutic target identification, uncovering resistance mechanisms, and surveillance of treatment response (Chaudhuri et al., 2017; De Rubis et al., 2019; Reinert et al., 2019; Kilgour et al., 2020; Lennon et al., 2020; Liu et al., 2020; Pantel and Alix-Panabières, 2024; Alix-Panabières and Pantel, 2025). Using LB in clinical practice would enable less invasive and more cost-efficient examinations for real-time monitoring of patients (Englmeier et al., 2023; Kramer et al., 2024; Liu S. et al., 2024). However, the sensitivity of current LB assays is limited by several factors, including the low and variable levels of tumor DNA shed into the bloodstream, which differ between patients and fluctuate over time (Connal et al., 2023; Menna et al., 2023). Therefore, high-sensitivity, high-specificity models integrating multimodal LB features need to be built and properly evaluated, along with their clinical utility.

In this regard, Artificial Intelligence (AI), including Machine Learning (ML) and Deep Learning (DL), can be highly helpful in handling the large amounts of complex data generated by the high-throughput biomarker platforms. Indeed, as single-candidate biomarker analysis is usually insufficient to predict diagnosis or prognosis (Fu et al., 2010; Burrell et al., 2013; Esposito et al., 2016), ML and DL enable simultaneous modeling of the effects of multiple biomarkers across different omics layers, accounting for their interactions. Consequently, integrating multiple biomarkers with AI can significantly improve the performance of LB tests in predicting various outcomes in cancer patients (Ko et al., 2018; Chen et al., 2023).

Here, we define multiomics integration as the process of combining molecular features originating either from different biological entities (e.g., ctDNA mutations and EV proteomics) or from different ‘omics’ layers within the same entity (e.g., ctDNA genomics and transcriptomics) into a single model. Although this integration typically involves tabular data, it aligns conceptually with multimodal ML, which combines diverse data types such as images (radiomics, pathomics), clinical reports, and time series. From this perspective, multiomics integration can be seen as a specific instance of multimodal learning in the biomedical context (Briscik et al., 2024). Multimodal integration builds on multimodal representation, translation, alignment, co-learning, and data fusion. Introducing these approaches to health data analysis is challenging, but they will be highly valuable and drive rapid advances in multiomics biomedical integration studies.

Our PubMed search identified relatively few studies integrating LB features across multiple molecular layers. This search, intended to identify representative studies rather than to constitute a systematic review, provided examples of current integrative approaches and their associated methodological challenges (Supplementary Table S1). The research was conducted via PubMed on research articles published with the terms (“cancer” OR “carcinoma” OR “tumor” OR “neoplasm”) AND “liquid biopsy” AND (“machine learning” OR “deep learning” OR “artificial intelligence”) AND (“early detection” OR “minimal residual disease” OR “prognosis”), excluding meta-analyses and reviews, and focusing on human studies. We conducted the search in January 2026 and included articles published from January 2020 to January 2026, yielding 144 records. Six authors screened the abstracts to identify studies integrating different types of LB features into a single model; 123 records were excluded at this stage. Three authors then reviewed the remaining articles in more detail. Four additional articles identified through reference lists and manual searching met the same selection criteria, resulting in 25 studies included in the descriptive analysis. Their characteristics are presented in Supplementary Table S1. Of these, 5 were prospective, and 18 were retrospective. These studies mainly reported early cancer detection outcomes (19 articles), including multi-cancer early detection (MCED). Methods for model development varied across studies, and only 6 performed external validation on a completely independent dataset or study (temporal, technical, geographical, or domain). The most recent studies implement this in their research. Neither calibration nor clinical utility was assessed in any of the studies. We used additional publications cited in the methodological discussion, including references from year 2026 identified after the search, to provide context; these were not included in these 25 studies or the corresponding descriptive counts. The limited number of studies identified may, at least partly, reflect the complexity of implementing AI-based integrative approaches in this setting (Liu et al., 2021).

Given the narrative scope of this contribution, the literature search was restricted to PubMed and predefined search terms and was not intended to provide an exhaustive or systematic characterization of the field. Consequently, relevant studies indexed in other databases or using terminology not captured by our search strategy may have been missed, and the relatively small number of integrative studies identified should not be interpreted as a definitive estimate of the volume of literature in this area. Instead, the search identified representative examples and methodological gaps in the application of AI-based integrative tools and model evaluation within the LB field. We therefore used these studies as representative examples throughout the manuscript to illustrate methodological challenges and current practices.

Several recent reviews have examined the application of AI to LB data, including assessments of diagnostic performance, early cancer detection, and disease-specific applications of multiomics integration (Eskandar, 2025; Filis et al., 2026; Sisca et al., 2026). The integrative studies identified in our search exhibited heterogeneous methodological practices and recurring gaps across stages of the analytical workflow. In contrast to evidence- or performance-focused reviews, this Methodological Article uses the available integrative LB literature as a basis for an end-to-end methodological framework covering study design and sample-size planning, preprocessing, dimensionality reduction and feature selection, multimodal fusion, model development and validation, interpretability, external validation and model updating, and clinical utility assessment. By linking these methodological considerations to common pitfalls and best-practice recommendations, we aim to provide a practical roadmap for developing robust, reproducible, and clinically translatable AI-based integrative LB studies. We conclude with an eight-point checklist summarizing these recommendations for designing, analyzing, and evaluating AI-based multimodal LB studies.

## Study purpose and design

### Study objectives and clinical context

As in any other study, the first step in designing an LB integration study is to define its primary objective. In this regard, LB biomarkers may benefit cancer patient management through early detection, TOO prediction, MRD monitoring, prognostication, therapeutic target identification, uncovering resistance mechanisms, and treatment response surveillance (Chen et al., 2023). Additionally, defining the necessary endpoints (primary and secondary) is crucial to ensure accurate and objective assessment. Poor selection of endpoints hinders the interpretation and implementation of the findings (McLeod et al., 2019). When assessing prognosis, the time from diagnosis to death (i.e., overall survival) is considered the primary endpoint for oncologic clinical trials. Other endpoints currently considered in prognostic studies include disease-specific survival, progression-free survival (measured as the time from diagnosis until the first evidence of disease progression or death), and disease-free survival (measured as the time from diagnosis until the first evidence of disease recurrence or death) (Delgado and Guddati, 2021). If the study’s purpose is (early) diagnosis, the presence of the disease would be the expected outcome.

### Study design and population

The research objectives, as well as the feasibility of conducting the study prospectively or retrospectively, determine its design. Supplementary Table S2 presents possible study designs related to each type of objective. Currently, many studies on LB are retrospective case–control studies that use already collected samples to measure LB biomarkers. This is the case for 18 out of the 25 studies identified in the literature search (Supplementary Table S1). This type of design lets researchers leverage prior efforts, making it less time-consuming and more cost-effective, though it may be subject to bias. Moreover, epidemiological/clinical data may be incomplete or inadequate in answering the research questions appropriately. In contrast, prospective studies would overcome these limitations by enabling real-time data collection under controlled conditions, thereby increasing the reliability and clinical relevance of LB biomarker validation.

Another key aspect of the study design concerns the definition of the population, including the selection criteria and the circuits required to identify the subjects. Selection criteria are described in 22 out of the 25 identified studies.

### Sample size

Moreover, estimating the number of individuals to recruit (i.e., the sample size) also affects model performance. Despite its importance, few studies mention this point (Dhiman et al., 2023). Only two papers from the bibliographic search estimated the sample size by conducting a power analysis after establishing a desired area under the curve (AUC) of 0.95 and 0.98 (Chabon et al., 2020; Wang et al., 2022). A method proposed by Riley et al. (2020) enables calculation of sample sizes for clinical prediction models with binary, time-to-event, and continuous outcomes, using a four-step procedure. Software for these calculations is available in the *pmsampsize package* (v1.1.3) in *Stata* or *R*. The package also estimates the minimum sample size required for external validation of clinical prediction models (Riley et al., 2021, 2022).

However, these strategies were primarily developed for conventional clinical prediction models and may not directly translate to high-dimensional ML or DL settings, where the effective model complexity depends not only on the number of measured features but also on signal strength and sparsity, feature selection or dimensionality reduction procedures, regularization, and the learning algorithm itself. Specific methods have been proposed for high-dimensional biomarker classifiers, showing that the required sample size can vary substantially according to these characteristics (McKeigue, 2019). Indeed, methodological evidence in oncology indicates that ML approaches may require substantially larger development samples than conventional regression models (Tsegaye et al., 2025). In practice, when a pilot or representative dataset is available, learning-curve or simulation-based approaches can provide a pragmatic strategy by repeatedly training and evaluating the complete analytical pipeline across increasing sample sizes, and assessing when predictive performance and model stability reach a prespecified target or show diminishing improvement (Dayimu et al., 2024). Recent methods extend this principle to high-dimensional transcriptomic data (Qi et al., 2025) and to selected ensemble ML algorithms (Mitsakakis et al., 2026), although no single approach is currently applicable across all multiomics data structures and model classes.

Importantly, sample-size planning for model development should be distinguished from the sample-size requirements for validating an already-trained model. In external or clinical validation, the objective is generally to estimate predictive performance with adequate precision rather than to provide sufficient data for model fitting (Riley et al., 2021, 2022). Methods such as Sample Size Analysis for Machine Learning (SSAML), available at https://github.com/GoldenholzLab/SSAML, provide model-agnostic estimates of the sample size required to achieve predefined precision and confidence for performance measures and can also accommodate complex ML and DL models (Goldenholz et al., 2023). Therefore, select the sample-size strategy based on whether the study aims to develop a new model or validate an existing one.

### Clinical, demographic and epidemiological information

Furthermore, the information to be collected in the study needs to be clearly defined. This includes demographic and epidemiological information, clinical characteristics of subjects, pathological or radiological tumor details, and patient follow-up. These traits can influence cancer detection, prognosis, biomarker levels, and therefore performance in outcome prediction. Specifically, age, gender, race, and ethnicity (Boffetta and Nyberg, 2003; Colditz and Wei, 2012; Bray et al., 2024) significantly impact the prevalence, progression, and response to cancer detection technologies (Colditz and Wei, 2012; Molina-Montes et al., 2020, 2021). Similarly, clinicopathological variables—including cancer type and stage, prior treatments, comorbidities, and genetic profile of both host and tumor—also affect the cancer prognosis (Weiss and Koepsell, 2014). Nguyen et al. (2023) also demonstrated the influence of clinical features on model predictions by evaluating the potential confounding effects of gender, age, cancer burden, and cancer stage in their integrative model for MCED. Collecting this information and incorporating these variables into AI-based modeling as potential confounders, effect modifiers, or mediators enhances the model’s validity and accuracy, making the results more robust, interpretable, clinically relevant, and generalizable to other populations (Molina-Montes et al., 2020, 2021). Another essential point in multicentric studies is standardizing the workflow for information collection. Validated questionnaires ensure reliable data, minimizing reliance on different criteria for variable definition and categorization. Furthermore, studies have demonstrated that sampling conditions influence biomarker discovery in LB (Batool et al., 2023). Therefore, information regarding biosample collection, processing, and storage is necessary to ensure that these factors do not influence the results.

## Characteristics of liquid biopsy features

LB enables the exploration of numerous tumor-derived biomarkers present in body fluids. Among these, CTCs—tumor cells shed from the primary tumor and/or from distant metastases into the bloodstream—are well studied (Pantel and Alix-Panabières, 2010). Beyond cellular components, LB allows the study of other circulating biomarkers, including cfDNA and cell-free RNA. When derived from the tumor, these are referred to as ctDNA and circulating tumor RNA (ctRNA), respectively. ctDNA consists of fragmented tumor-derived DNA found in the bloodstream (Wan et al., 2017), while ctRNA corresponds to RNA molecules released by tumor cells (Stejskal et al., 2023). Additionally, microRNAs (miRNAs), small non-coding RNAs involved in gene regulation, and various circulating proteins, including those studied through secretomics (the analysis of secreted proteins), represent important analytes examined through LB. Other tumor-induced circulating cell types, such as circulating immune cells, circulating endothelial cells, cancer-associated fibroblasts, and TEPs, are also gaining increasing attention in oncological research and can be analyzed through LB. EVs, which carry molecular cargo such as proteins, RNA, and DNA, are another relevant biomarker class. Among them, exosomes (Van Niel et al., 2018), small EVs ranging from 30 to 150 nm and formed within the endosomal system, are widely studied. These circulating components have diverse cellular origins, which researchers must consider when addressing specific biological questions in cancer research. Moreover, researchers can assess them through various analytical approaches, including measuring abundance or concentration, and characterizing molecular profiles using high-throughput technologies. Such analyses depend on both the origin and composition of each component. Figure 1 illustrates these relationships, which are essential for determining variable types in multiomics integration studies. These distinctions also inform the statistical properties of the derived features and guide the selection of appropriate data processing steps. Further details are provided in Supplementary Table S3.

**Figure 1:**
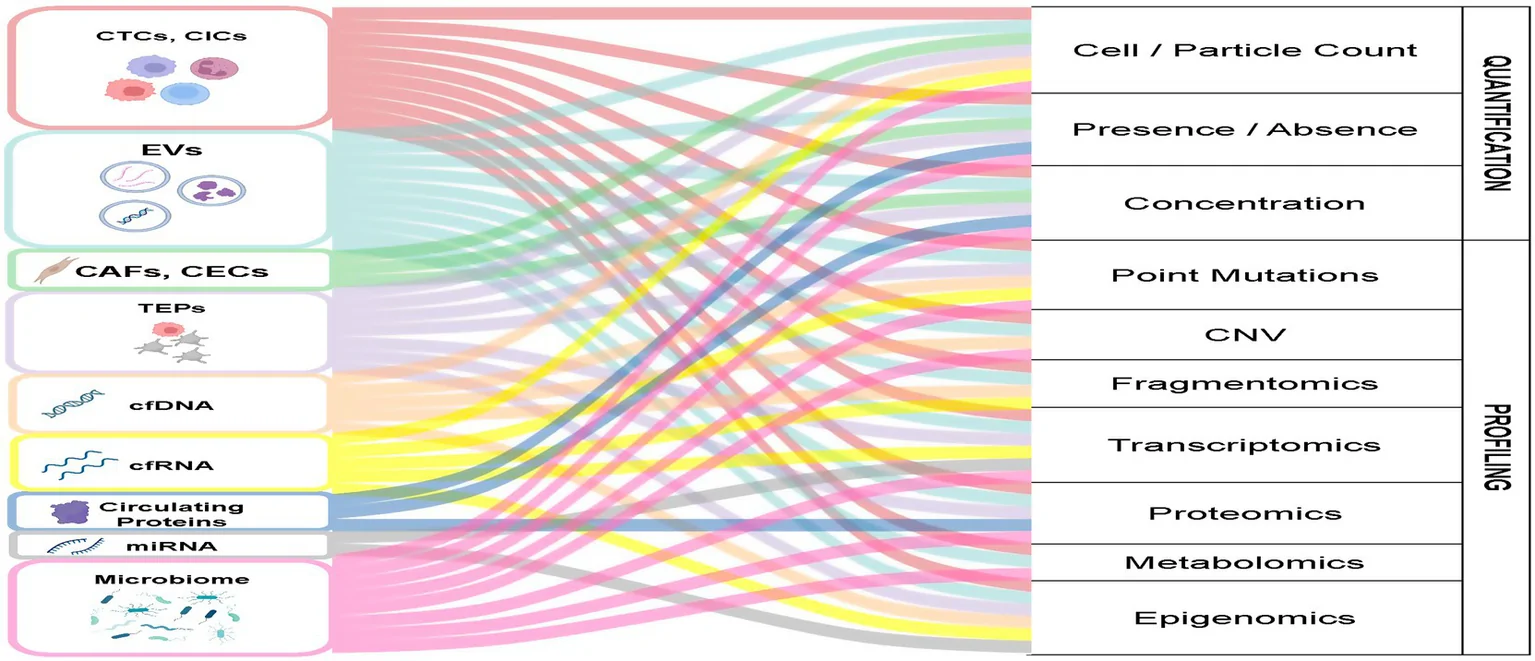
Correspondence between liquid biopsy components and omics layers/features. Liquid biopsy components can be characterized through quantification-based measurements, including cell/particle counts, presence/absence, and concentration, or through molecular profiling, including point mutations, copy-number variation, fragmentomics, transcriptomics, proteomics, metabolomics, and epigenomics. The diagram illustrates the types of measurements and molecular profiles that can be derived from different circulating components and subsequently considered for multiomics integration. CTCs, circulating tumor cells; CICs, circulating immune cells; EVs, extracellular vesicles; CAFs, cancer-associated fibroblasts; CECs, circulating endothelial cells; cfDNA, circulating free DNA; miRNA, microRNA; CNV, Copy number variation.

## Data preprocessing

### Data exploration, data cleaning and outlier handling

Understanding and visualizing data characteristics, also known as data exploration, is an initial step in data preprocessing and is particularly important for creating a robust dataset before modeling multimodal LB features using AI. This process is frequently followed by a data cleaning step that detects and corrects inconsistent records in the dataset. This step should be performed for each LB modality type and clinical/epidemiological variable. Outlier handling should also be considered at this point.

### Missing data and imputation

Missing data and incomplete records are another common challenge in multimodal integration. Imputation methods can complete records, enabling effective training of AI models. *MissForest (missForest v1.6.1)* is a popular imputation method that can handle any type of data, including those with complex relationships between variables (Stekhoven and Bühlmann, 2012). It is a non-parametric method based on random forests (RF), which achieves high accuracy and performs well on small datasets but is highly computationally demanding. K-nearest neighbors (KNN) imputation models are also frequently used for numerical data imputation (Troyanskaya et al., 2001), and require no training. They perform well for small datasets while preserving the original data distribution. Multiple Imputation by Chained Equations (MICE) generates multiple imputed datasets to pool the results of the developed model, allowing for the incorporation of uncertainty associated with the imputed variables’ actual values (Austin et al., 2021). This method applies to various data types, but it requires parameter tuning and becomes computationally intensive with many variables. Multiple imputation can be performed with the *mice* package (v3.18.0) in *R*. Wang et al. (2025) used MICE to impute data followed by sensitivity analysis. Although many studies perform imputation, very few assess the quality of the imputation itself or its impact on model performance, given the distribution of missing data.

Importantly, missing values within an available data layer should be distinguished from missing modalities, where an entire omics assay is unavailable for some patients. This may result from limited sample material, assay failure, or differences in which tests were performed, and restricting analysis to patients with all modalities can substantially reduce the sample size. An absent layer can be reconstructed from observed data; for example, Voillet et al. (2016) used multiple imputation to replace entire missing rows in omics data tables before multiple factor analysis. However, reconstructing a whole unmeasured layer relies on assumptions about relationships between modalities and about the mechanism underlying modality missingness. In particular, approaches relying on ignorability assumptions are generally more defensible under missing completely at random (MCAR) or missing at random (MAR) than under missing not at random (MNAR) (Flores et al., 2023). When feasible, models that can natively accommodate missing modalities may therefore be preferable to reconstructing entirely unobserved data layers, as they avoid relying on potentially strong cross-modal imputation assumptions (Nguyen and Vafaee, 2026). The extent and reasons for modality missingness should therefore be reported, and the effect of reconstruction, or of using models designed to operate with incomplete modality sets, on downstream predictions should be evaluated.

Furthermore, zeros in the measures can be biological (truly absent from the data) or technical (introduced by the detection limit or library preparation steps) (Silverman et al., 2020). Therefore, a lack of biomarker detection does not necessarily imply its absence in the sample. Importantly, zero handling should be guided by the mechanism generating the zero and the statistical structure of the corresponding LB feature (Figure 2). True biological or structural zeros should generally be preserved, whereas values resulting from detection limits, insufficient sampling, or technical dropout may require specific treatment. For continuous assays with a defined limit of detection, non-detects should be considered as censored measurements rather than automatically interpreted as true absence (Herbers et al., 2021). In sequencing-based data, technical zeros may arise from insufficient capture, amplification, or sequencing depth (Jiang et al., 2022), while compositional data require particular attention because zero values preclude log-ratio transformations (Quinn et al., 2019). When zero replacement or imputation is required, the selected method should respect the statistical properties of the corresponding data type, and the robustness of the results to alternative zero-handling strategies should be evaluated through sensitivity analyses (Sterne et al., 2009). Several zero-imputation approaches have been developed for specific data structures, including *DrImpute* (v1.0) and *scImpute* (v0.0.9) for single-cell RNA-sequencing data, LLSimpute (implemented as *llsImpute* in *pcaMethods* v2.4.0) for microarray-like expression data, and multiplicative replacement methods implemented in *zCompositions* (v1.6.2) for compositional datasets. These methods rely on different assumptions and should therefore be applied only when those assumptions match the corresponding LB data layer.

**Figure 2:**
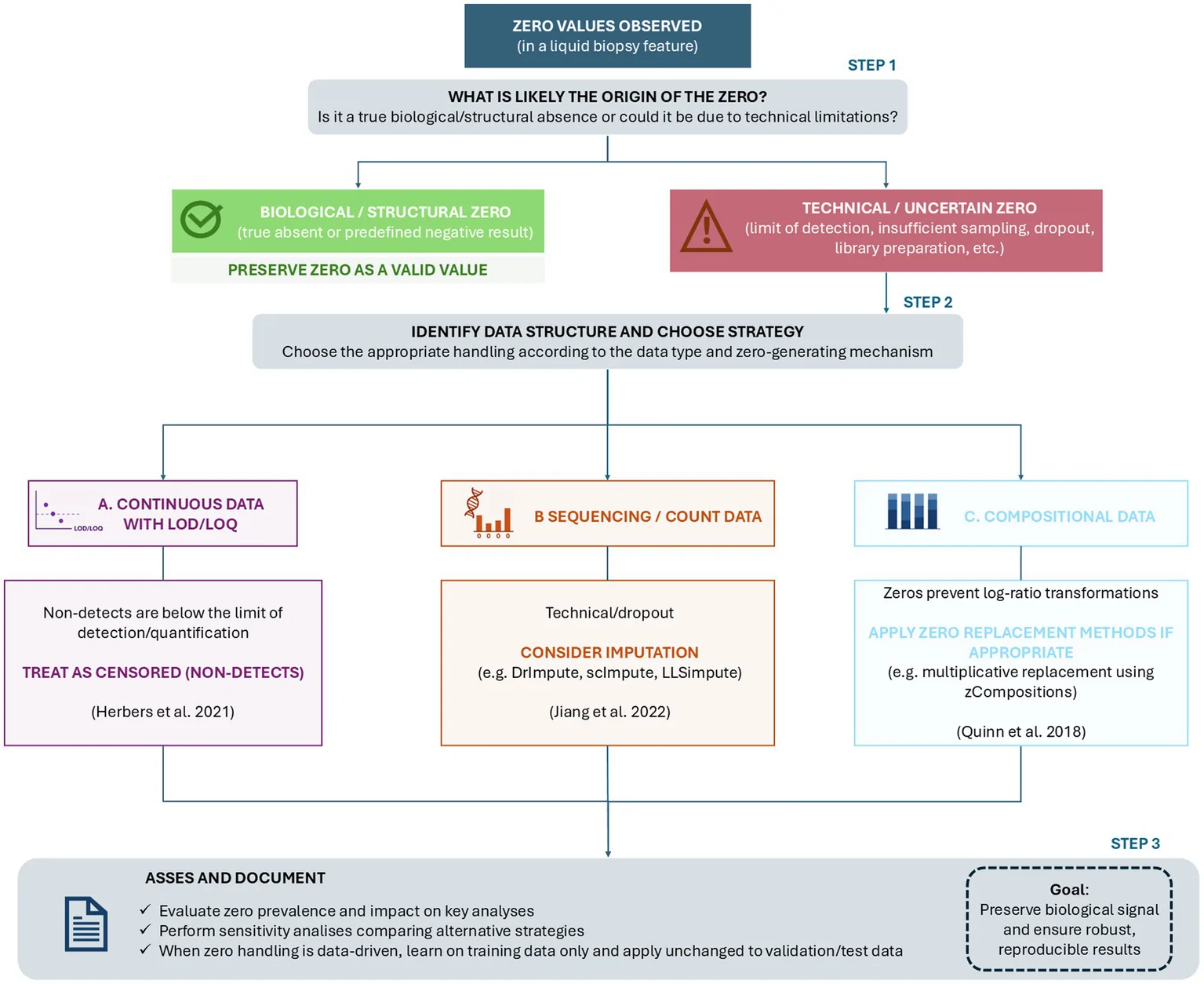
Decision flowchart for zero handling in liquid biopsy datasets. The workflow comprises three steps: (1) determining whether an observed zero represents a true biological or structural absence or arises from technical limitations; (2) selecting an appropriate handling strategy according to the data structure and the underlying zero-generating mechanism; and (3) assessing and documenting the impact of the selected strategy to ensure robust and reproducible results. True biological or structural zeros should be preserved, whereas technical or uncertain zeros may require treatment as censored observations, imputation, or zero replacement for continuous, sequencing/count, and compositional data, respectively. LOD, limit of detection; LOQ, limit of quantification.

### Batch-effect correction and data normalization

Even within the same data type, variability in biosample collection, processing, storage, or lab protocols—especially in retrospective studies—can introduce inconsistencies that compromise data reliability and comparability (Batool et al., 2023). Addressing potential batch-effects is therefore essential to minimize technical variation in downstream analyses. Supplementary Table S4 describes various batch-effects correction methods, along with a brief description of the input data type, their advantages, and disadvantages.

When batch-effects correction forms part of a predictive workflow, its application to validation samples requires particular care. Estimating batch-effects using both training and held-out samples can introduce information from the evaluation set into model development, a form of data leakage. Methods should therefore be selected with their intended use on new samples in mind. Frozen surrogate variable analysis (fSVA), for example, learns adjustment information from a reference training set that can subsequently be applied to new samples (Parker et al., 2014). *ComBat* can also align observed batches to a designated reference batch, but specifying a reference batch alone does not establish how samples from a previously unseen batch will be corrected (Zhang et al., 2018). More generally, batch-effect correction parameters and any reference data used for their estimation should be defined within the training framework and applied to validation data without re-estimation using held-out samples. Performance should then be assessed on appropriately held-out data. Box 1 summarizes this requirement as part of the broader principle that preprocessing must not use information from held-out data.

Data normalization is also a standard preprocessing step that scales data to improve consistency. The nature of each layer must be considered when normalizing data from an LB study that aims to integrate information across multiple features. As depicted in Supplementary Table S3, most LB biomarkers are binary or continuous variables, with varying ranges. Methods such as Z-score standardization, max-min normalization, log-scaling, and decimal scaling have been proposed for normalizing LB features (Liu et al., 2021).

### Dimensionality reduction and feature selection

All selected papers from our search considered more than one omics layer (analytes) containing multiple features. Hence, as high-dimensional data often contain correlated or redundant information (Pinsky and Zhu, 2011; Bansal and SullivanPepe, 2013; Demler et al., 2013) and can be challenging to model with limited sample sizes (McKeigue, 2019), dimensionality reduction and feature selection may be considered at both single- and multiomics levels.

There are unsupervised and supervised methods for dimensionality reduction. For single omics, PCA (Principal Component Analysis), Independent Principal Component Analysis, UMAP (Uniform Manifold Approximation and Projection), and t-SNE (t-distributed Stochastic Neighbor Embedding) are unsupervised methods, while Linear Discriminant Analysis (LDA) is a supervised dimensionality reduction method. For multiomics data, unsupervised methods include Canonical Correlation Analysis, MOFA (Multiomics Factor Analysis), or Joint and Individual Variation Explained, among others. Supervised methods include Partial Least Squares, Multivariate Integration, or DIABLO (Data Integration Analysis for Biomarker discovery using Latent Variable approaches for Omics studies). The *mixOmics* (Rohart et al., 2017) package (v6.36.0) in *R* offers many of the previously mentioned statistical methods for dimensionality reduction. Moreover, autoencoders or other DL methods are also suitable for this purpose.

Feature selection aims to identify the most informative and relevant biomarkers, improving model performance and interpretability. Feature selection techniques are categorized into filter, wrapper, and embedded methods (Saeys et al., 2007). Filter methods evaluate feature importance based on intrinsic properties. They include Pearson’s correlation coefficient, the F-statistic, Mutual Information, and the Chi-Squared statistic. Wrapper methods generate feature subsets using search algorithms. Strategies such as recursive feature elimination, sequential selection algorithms, and metaheuristic algorithms can be employed (Kohavi and John, 1997). For instance, the DEcancer framework (Halner et al., 2023) employed recursive feature elimination, guided by RF variable importance, to select a parsimonious multimodal biomarker panel that integrates protein biomarkers and ctDNA mutation features. This approach explicitly aimed to reduce redundancy while preserving predictive performance, highlighting the importance of systematic feature selection when combining heterogeneous LB modalities. Embedded methods perform feature selection during the model training process. These methods are specific to the adopted ML algorithm. Regularization methods like least absolute shrinkage and selection operator (LASSO), Ridge, and Elastic Net (ENET) were used for feature selection in some selected papers (Nguyen et al., 2023; Pham et al., 2023; van Eijck et al., 2024). Tree-based methods, such as RF and gradient boosting, were also considered to rank the importance of various biomarkers (Nguyen et al., 2023; Pham et al., 2023; Eledkawy et al., 2024). These methods perform feature selection and outcome prediction simultaneously.

## Integrative modeling

This phase comprises four important and complex steps: 1. multimodal fusion, 2. model tuning and internal validation, 3. model interpretability, and 4. external validation and model updating. Below, we describe each step and provide lists of tools for the reader to consider as they undertake this endeavor.

### Multimodal fusion

Also known as data fusion, it is essential when building integration models that combine information from multiple modalities (in this case, omics) to predict an outcome. Baltrusaitis et al. (2019) classified fusion techniques into model-based and model-agnostic approaches. The former models specifically address fusion in their construction and include Multi-Kernel Learning, Graphical Models, (NN), and Multi-View Learning, all of which encompass data fusion. The latter performs data fusion independently of the model used. Moreover, approaches can be divided into early, late, and intermediate fusion. Early fusion (feature-level fusion) refers to integrating all omics-derived features immediately after extraction, which can be achieved through concatenation, operations, or DL models. In late fusion (model-level fusion), models are built for each omics data layer and then integrated to produce a combined model output. More complex methodologies include intermediate fusion (also known as middle fusion or joint integration), in which individual models first analyze different layers before combining their outputs into a final prediction. Figure 3 further illustrates these terms. There are also mixed scenarios (mixed or hybrid fusion) where approaches from the two strategies are combined (Krones et al., 2025).

**Figure 3:**
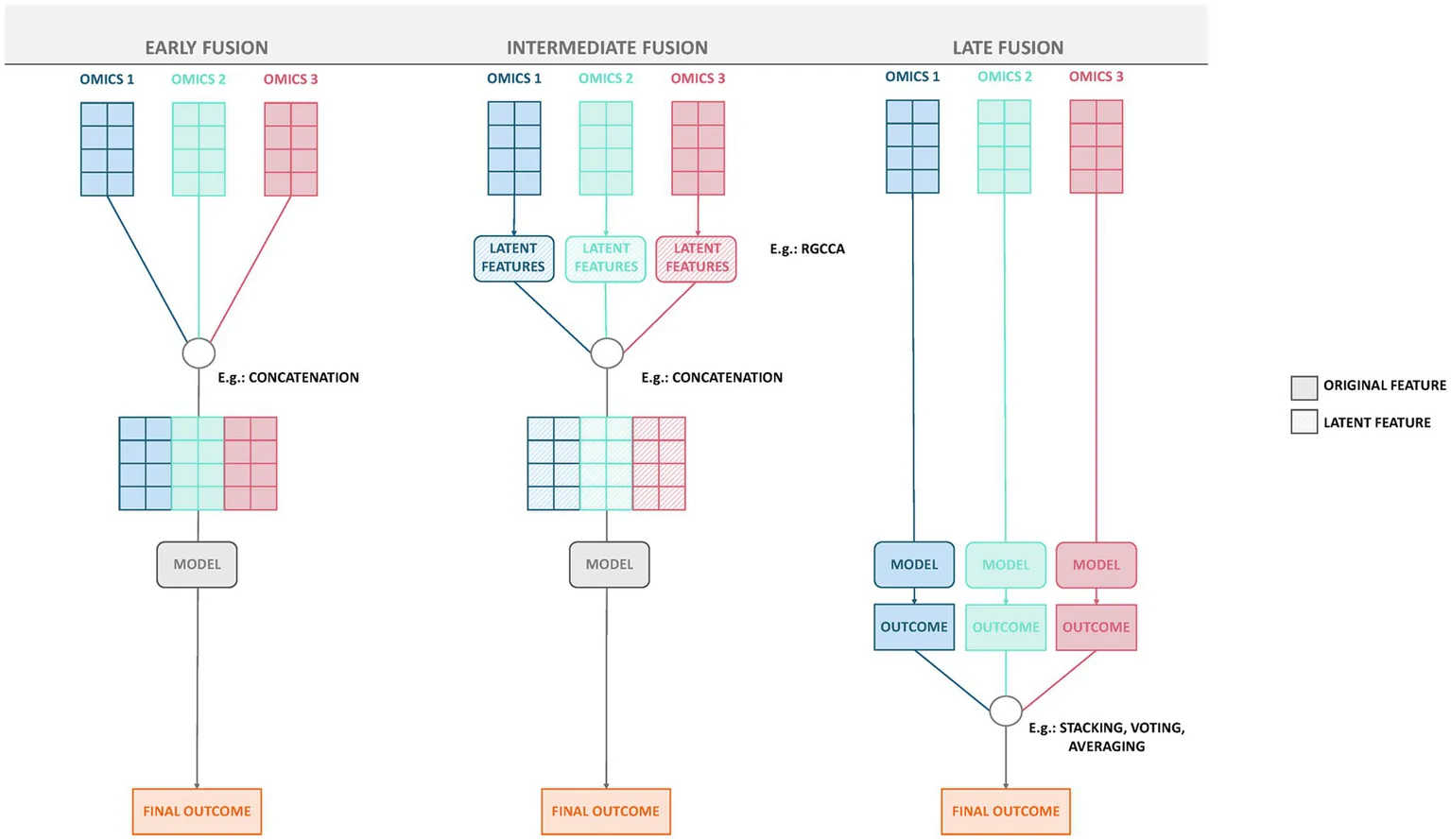
Data fusion approaches. Early fusion combines features from different omics layers before model fitting, for example, through feature concatenation. Intermediate fusion first derives latent representations from each omics layer and subsequently combines them before model fitting. Late fusion builds separate models for each omics layer and combines their predictions to obtain the final outcome, for example through stacking, voting, or averaging. RGCCA, Regularized Generalized Canonical Correlation Analysis.

To illustrate the aforementioned terms, we consider the methodological approaches adopted in Nguyen et al. (2023). Their MCED approach followed two strategies: first, they generated a single model using raw data from the LB feature modalities (targeted methylation, genome-wide methylation, ctDNA copy number alterations, fragment length, and end motifs), then evaluated three different ML approaches on the complete dataset. This is an example of an early fusion strategy that concatenates the individual features. Second, they built an ensemble stacking model that combines the outputs of nine base models (one per LB layer) with Linear Regression (LR). This is considered late fusion. For the first case, Extreme Gradient Boosting (XGBoost) outperformed the stacking ensemble model (Nguyen et al., 2023). This highlights the need to study different data fusion approaches, which can be decisive in improving model performance alongside the selected AI model.

Modality availability also affects the choice of fusion strategy. Conventional early fusion based on concatenating all feature blocks requires a complete input for each patient, unless missing blocks are reconstructed or the model is specifically designed to use an availability mask. Some intermediate-fusion methods can learn a joint representation from the modalities available for each patient (Lee and van der Schaar, 2021), but this capability must be established for the particular method rather than assumed for intermediate fusion in general. Late fusion can combine predictions from the modality-specific models that are available for a given patient, with combination weights adjusted to that subset of modalities.

Finally, it is important to consider which ML/DL algorithm performs the best. LR, especially with regularization, offers built-in feature selection and interpretability. Kernel-based methods address high dimensionality and have been used, for instance, to integrate proteomics and transcriptomics in predicting pancreatic cancer progression during chemotherapy (van Eijck et al., 2024). Decision trees and ensemble methods like RF and boosting algorithms (e.g., XGBoost, AdaBoost) are also widely applied for their balance of performance and interpretability. Support Vector Machines handle complex decision boundaries through kernels. In more complex settings, artificial NN can model nonlinear relationships but require larger datasets. Within this group, Multi-Layer Perceptrons have outperformed simpler models in certain multiomics applications, such as lung cancer classification (Kwon et al., 2023).

Other DL approaches, such as convolutional NN, attention-based models, and recurrent NN or long short-term memory networks (for longitudinal data), are emerging as powerful tools for integrating diverse omics layers. Transformers can integrate heterogeneous signals into a unified representation, using attention layers to highlight which molecular features or modalities carry the most predictive power for the task. In the LB context, transformer-based models have been applied directly to cfDNA fragments for representation learning and cancer prediction [e.g., DECIDIA (Liu et al., 2024b)]. Besides, in recent years, large language models (LLMs) have emerged as a breakthrough in AI, leading to widely known tools such as ChatGPT. These models can understand and generate human-like text by learning patterns from vast amounts of data. Moreover, foundation models are large-scale ML models that are trained on broad, general-purpose data (such as text, images, or code) and can be adapted to a wide range of downstream tasks. LLM-based foundation models, such as LLaMA, have already been fine-tuned in the LB setting to predict cancer from cfDNA (Liu et al., 2024a). While they have not yet been applied to LB multiomics integration, these approaches hold considerable promise for future applications. However, their broader adoption is currently limited by the relatively small sample sizes typical of multiomics LB studies, the substantial computational resources required to train or fine-tune large models, and the limited availability of large, diverse, and representative datasets. Therefore, their potential advantages over established ML approaches for LB multiomics integration remain to be demonstrated.

### Model tuning and internal validation

Model tuning is essential for selecting the optimal hyperparameters and model architecture (in DL). This process typically involves splitting the dataset into training, validation, and test sets. The model is trained on the training set, while the validation set is used for feature selection, hyperparameter optimization, architecture selection (in DL), and model selection (when several strategies are performed) (Figure 4). The final model is then evaluated on an independent test set, a step often referred to as internal validation, which assesses the model’s generalizability within the available data and ensures reproducibility. When traditional data splitting is not feasible because of limited sample size, resampling methods such as bootstrapping and k-fold cross-validation provide more robust performance estimates by repeatedly training and validating the model on different data subsets. In small-n/high-p settings, which are common in LB studies, nested cross-validation (an approach using an inner loop for model selection and an outer loop for performance estimation) may be required to avoid optimistic bias when feature selection or hyperparameter tuning is performed.

**Figure 4:**
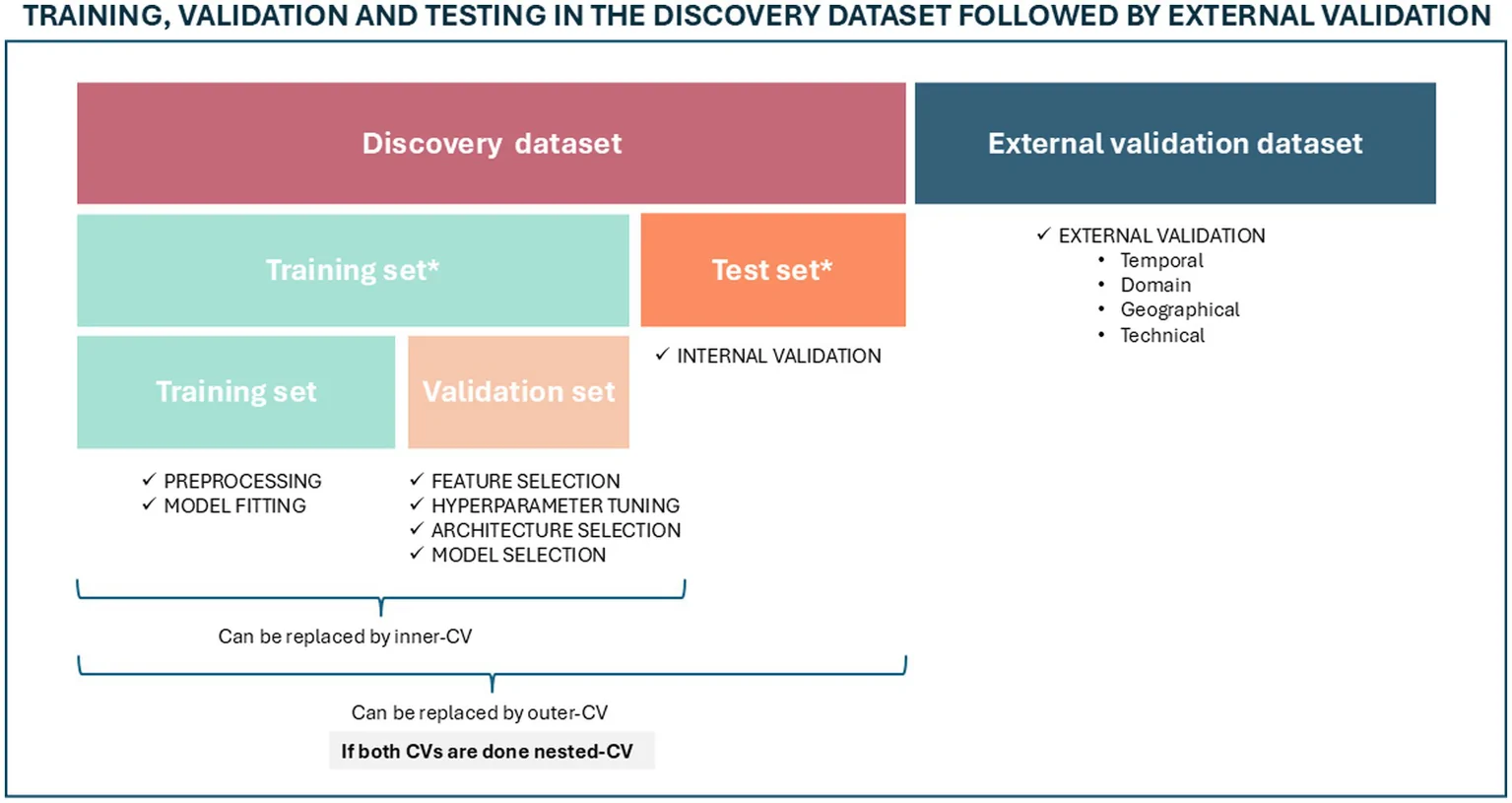
Schematic representation of data partitioning for AI model evaluation: includes training, validation, and test in the discovery dataset, followed by external validation on an independent dataset. The discovery dataset is partitioned into training, validation and test sets for model development and internal validation, followed by external validation using an independent dataset. Cross-validation (CV) can replace fixed validation and test partitions, with nested CV combining inner and outer loops for model tuning and unbiased performance estimation. External validation may assess temporal, geographical, domain or technical generalizability.

When late integration strategies are used, additional care is needed, as both the unimodal models (each with its own parameters) and the integration mechanism must be optimized. In such cases, model development should ensure that tuning of unimodal components and the final integration layer does not involve using data in the test set.

Class imbalance may be addressed during model tuning through class weighting or resampling methods such as the Synthetic Minority Over-sampling Technique (SMOTE) (Chawla et al., 2002). If resampling is used, it should be restricted to the training portion of each validation split; validation and test sets should retain their original class distribution. Because resampling alters the effective class prevalence seen during training, it can affect the calibration of predicted probabilities even when discrimination is preserved or improved. Model performance should therefore be evaluated on unresampled data, with both discrimination and calibration assessed explicitly. In clinical prediction settings, oversampling methods including SMOTE have been reported to produce poorly calibrated and potentially overestimated predicted risks when probability estimates are interpreted directly (Piccininni et al., 2024).

Reproducibility should also be considered throughout model development. Studies should report the software and package versions, random seeds, relevant preprocessing and feature-selection settings, and the hyperparameter configurations and search spaces evaluated during model tuning. For stochastic algorithms, reporting these elements is particularly important to facilitate independent replication and comparison across studies.

Two critical measures of model validation are discrimination and calibration (Steyerberg, 2019). These metrics provide unique insights into classification or regression performance, and the choice of metric depends on the specific application and the nature of the data. This ensures a comprehensive evaluation of models, leading to more accurate and reliable predictions. Discrimination measures the model’s ability to distinguish between different outcome states, such as the presence or absence of cancer. It evaluates how well the model distinguishes individuals with various outcomes based on their predicted risk scores. We have summarized various classification assessment methods in Supplementary Table S5 (Tharwat, 2018), highlighting their methodologies, strengths, weaknesses, and applicability. One example of their application is proposed by Eledkawy et al. (2024), who assessed the precise detection and classification of cancer using LB data and advanced ML techniques to analyze ctDNA mutations and protein biomarker concentrations. To evaluate the proposed model, they computed precision, recall, F1-score, accuracy, and AUC, showing the receiver operating characteristic (ROC) curve and confusion matrix results (Eledkawy et al., 2024)^,^ and comparing them with those from previous methods on the same dataset.

This example also illustrates why discrimination metrics should account for class imbalance. When cancer cases are rare, a high ROC-AUC can coexist with a low proportion of true cases among positive predictions. Precision–recall (PR) curves and PR-AUC can therefore complement ROC-AUC, provided you report the proportion of cases in the evaluation cohort, as it influences precision and PR-AUC. Interpret these results in the context of the prevalence expected in the intended clinical setting.

On the other hand, calibration refers to the agreement between predicted probabilities and actual outcomes. A well-calibrated model provides accurate probability estimates across the entire range of predictions, ensuring that the predicted risk corresponds closely to the actual risk observed in the population (Steyerberg et al., 2010). Calibration is crucial in clinical settings, where precise risk estimates can influence treatment decisions and patient management strategies. Standard metrics for assessing calibration include the observed-to-expected (O/E) ratio, calibration-in-the-large, calibration slope, and calibration plots. The O/E ratio compares the observed number of events to the expected number of events. Conversely, calibration-in-the-large evaluates the overall agreement between predicted probabilities and observed outcomes by adjusting the model intercept to correct for systematic over- or under-prediction (Steyerberg et al., 2010; Van Calster et al., 2019). The calibration slope assesses whether the predicted probabilities are too extreme or not extreme enough (Steyerberg et al., 2010; Van Calster et al., 2019). Finally, calibration plots compare predicted probabilities with observed outcomes, including the information on calibration-in-the-large and the calibration slope. These plots are useful for visually assessing how well the predicted probabilities align with actual outcomes across different risk levels (Steyerberg et al., 2010).

Additionally, the Brier score provides a single measure of the accuracy of probabilistic predictions by computing the mean squared difference between predicted probabilities and actual outcomes, capturing both calibration and discrimination aspects (Brier, 1950).

For time-to-event outcomes, validation must account for follow-up time and censoring rather than treating all patients without an observed event as event-free. Discrimination can be assessed using the concordance index (C-index) and, at prespecified clinically relevant time horizons, time-dependent AUC. Calibration should assess agreement between predicted and observed event probabilities at the same time horizons, for example using calibration plots and appropriate summary measures. When a competing event precludes the outcome of interest, both risk prediction and validation should account explicitly for competing risks rather than treating competing events as non-informative censoring.

### Model interpretability

After selecting the best LB integrative model, evaluating its interpretability is crucial, especially in healthcare, where understanding decisions is key for biological interpretation and clinical implementation. Experts must fully understand the model’s pros and cons to translate them effectively into clinical practice.

For instance, consider building a model using proteomics and metabolomics data to predict disease recurrence. We could apply: (1) interpretable models offering transparency by design, as their decision rules or coefficients can be directly evaluated, being linear and logistic regressions, generalized linear models, generalized additive models, decision trees, and decision rules, the most interpretable ones; or (2) more complex models, some of which are seen as black boxes, where interpretability methods must be implemented. After achieving high accuracy with the chosen model, it becomes crucial to understand which biomarkers are driving predictions. For the first group of models, interpretability is straightforward. For the most complex models, we could apply model-agnostic methods, which are flexible and work with any model and can be classified as global or local (Molnar, 2025). Global model-agnostic methods provide insights into a model’s overall logic, while local methods explain the rationale behind specific predictions. For instance, SHAP (SHapley Additive exPlanations) is a model-agnostic local interpretation method that estimates the contribution of each feature to the output. This analysis may reveal that proteomic features in our example strongly influence predictions, highlighting key biomarkers and improving the model’s clinical relevance and cost-effectiveness. In fact, Wang et al. (2025) used SHAP values to rank model features, finding MRD, treatment modality, and baseline ctDNA status as the top 3 contributors of their NN-based PRIME model (Progression Risk prediction using Interpretable ML on ctDNA-based genomic Mutations, MRD, and clinical-therapeutic features). A summary of the agnostic methods is presented in Supplementary Table S6.

Interpretability in integrative LB models nevertheless requires particular caution. In high-dimensional multiomics settings, the large number of potentially correlated and redundant features within and across omics layers can complicate feature-level attribution and its biological interpretation (Toussaint et al., 2023). In particular, correlations among features can affect SHAP-based attribution, potentially distributing or misassigning importance among related predictors (Hu et al., 2024). Therefore, interpret individual feature-importance values in the context of correlated biomarkers and, when appropriate, complement them with analyses at the pathway, feature-group, or omics-modality level. Importantly, feature attribution reflects the contribution of a variable to a model prediction and should not, by itself, be interpreted as evidence that the corresponding biomarker is a causal driver of the outcome (Carriero et al., 2025). Highly ranked features may instead represent correlated proxies, confounded associations, or technical effects. Consequently, interpretability analyses in LB multiomics should primarily be hypothesis-generating: biomarkers or pathways showing robust and reproducible importance can be prioritized for replication in independent cohorts, orthogonal validation using alternative measurement approaches, and, when biologically appropriate, functional experiments before drawing mechanistic or clinical conclusions.

### External validation and model updating

External validation is a cornerstone of developing robust integrative LB models. This process ensures the model generalizes effectively to new, independent data and related settings (Binuya et al., 2022). This type of validation is critical for ensuring the model’s applicability in real-world clinical environments. It can take several forms and is implemented on an external validation dataset (Figure 4). Temporal validation assesses the model’s performance on data collected in a distinct time period from the training set, ensuring its consistency and robustness over time. Geographic validation uses data from multiple locations to test the model’s adaptability to diverse patient populations and healthcare practices. Domain validation involves applying the model across different clinical settings, such as transitions from secondary to primary care, to evaluate its broader utility. Finally, technical validation ensures reproducibility across platforms and robustness under varying technical conditions. Six of the selected papers in this review validated or partially validated their results using distinct methodologies (Supplementary Table S1), with this trend increasing in recent years. Chabon et al. (2020) externally validated their *LungClip* model, which discriminates early-stage lung cancer patients from risk-matched controls, in a prospectively collected independent cohort. Eledkawy et al. (2024) partially validated their results using an external database, adapting their approach to the available features. As with internal validation, models are evaluated externally using discrimination and calibration metrics. Often overlooked due to the scarcity of validation data, this step is fundamental to model generalizability.

An external validation dataset should be appropriate for the intended use of the model and representative of its target population and clinical setting (Riley et al., 2024). It should be independent of the development data, follow eligibility criteria and outcome definitions consistent with the intended use of the model, and contain all predictors required to generate predictions, measured at the appropriate time point and using procedures representative of those expected in practice (Riley et al., 2024). In LB studies, particular attention should also be paid to differences in pre-analytical procedures, assay platforms, laboratory or batch conditions, and patterns of missingness, as these factors may introduce technical variation in addition to differences in the underlying population (Batool et al., 2023). Finally, the validation dataset should have an adequate sample size to estimate relevant performance measures with sufficient precision, including discrimination, calibration, and, when appropriate, clinical utility (Riley et al., 2021).

When interpreting external validation results, differences in performance should not automatically be attributed to model overfitting. A structured assessment should first compare the development and validation populations in terms of case mix, outcome prevalence or incidence, predictor distributions, and, particularly for LB data, relevant technical and measurement characteristics (Debray et al., 2014). Model performance should then be evaluated across complementary dimensions, including discrimination, calibration-in-the-large, calibration slope and calibration plots, as well as clinical utility when appropriate (Collins et al., 2024). Preserved discrimination accompanied by a shift in calibration-in-the-large may indicate a change in baseline outcome risk, whereas deterioration of the calibration slope may reflect overly extreme predictions or changes in predictor–outcome associations (Collins et al., 2024). Conversely, reduced discrimination in the presence of substantial case-mix or technical differences may reflect population or measurement shift rather than overfitting alone (Debray et al., 2014). A marked discrepancy between internal and external performance in otherwise comparable populations may increase concern about optimism or overfitting during model development (Collins et al., 2024). These mechanisms are not mutually exclusive and should therefore be investigated jointly rather than inferred from a single performance metric (Binuya et al., 2022).

When external validation reveals inadequate performance, model updating may be considered to adapt the model to the target population or setting. Updating may also be needed as additional patients or data become available, new biomarkers are identified, clinical practices evolve, or the model is implemented in different centers or populations. Such changes may alter the predictor distributions or the outcome prevalence, leading to calibration drift or reduced generalizability over time. The extent of updating (from recalibration to model revision or redevelopment) should be guided by the type and magnitude of performance deterioration, the nature of the new information, and the amount of data available for updating (Riley et al., 2024). When discrimination is preserved, but systematic miscalibration is observed, relatively conservative approaches may be sufficient. Recalibration-in-the-large adjusts the intercept or baseline hazard to account for differences in baseline risk, whereas recalibration of both the intercept and calibration slope additionally adjusts the spread of predicted risks. When predictor–outcome associations differ substantially between development and target populations, more extensive model revision, including re-estimation of some or all predictor effects, may be required. Model extension can additionally incorporate new predictors when relevant information is available in the target setting (Binuya et al., 2022). Because increasingly extensive updating requires estimating more parameters and therefore larger sample sizes, the least complex updating strategy that adequately addresses the identified need should generally be preferred to reduce the risk of overfitting (Vergouwe et al., 2017). Importantly, performance estimates based on the same data used for model updating are optimistic; therefore, the updated model should undergo appropriate internal validation and, ultimately, further external validation before clinical implementation.

## Clinical utility and economic impact evaluation

Clinical utility is a critical aspect when evaluating LB integrative models. The goal of any predictive model in healthcare is not just to produce accurate predictions but to positively impact clinical outcomes and patient management. It measures how well a model aids in clinical decision-making, improves patient outcomes, and is cost-effective in real-world settings (Binuya et al., 2022), justifying its use over existing methods.

Impact studies evaluate the model’s actual effect on clinical practice. They can be designed as randomized controlled trials, before-and-after studies, or other comparative studies that measure changes in clinical outcomes, patient behaviors, and healthcare utilization following implementation of the predictive model (Collins et al., 2015). Moreover, the model’s potential impact can be assessed using several metrics that, although often discussed, few publications have measured in their work. The net benefit quantifies clinical benefit, penalizing true positive classifications with false positive classifications (Vickers et al., 2016), as shown in Equation 1:


$$\mathrm{Net}\;\text{Benefit}=\frac{\mathrm{TP}}{n}-\frac{\mathrm{FP}}{n}(\frac{p_{t}}{1-p_{t}})$$ (1)

Where TP: True Positives, FP: False Positives, n: Total number of patients, p_t_: threshold where the expected benefit of treatment is equal to the expected benefit of avoiding treatment.

Decision curve analysis (DCA) evaluates the net benefit of a predictive model across different threshold probabilities (risk levels at which a clinician would choose to intervene), identifying when the model provides more benefit than harm compared to default strategies. The probability threshold is conceptually related to the minimum positive predictive value (PPV, the proportion of individuals with a positive test who truly have the disease) for a test to be clinically useful. PPV critically depends on the disease prevalence in the screened population, as well as on test sensitivity and specificity. To illustrate these concepts, consider an AI-based LB model developed for early cancer detection, showing an AUC of 0.90 (high discrimination). A clinical deployment of this test would require selecting an operating point aligned with the intended use. If we want to use this AI model for population-level screening, a very high specificity threshold may be prioritized to minimize false positives and unnecessary downstream diagnostic procedures. Considering an operating point with 95% specificity and 60% sensitivity and a disease prevalence of 1% in the population to be screened, the AI model would identify approximately 6 true positives and 50 false positives per 1,000 screened individuals, which represents a substantial downstream diagnostic burden despite the high AUC of the AI model.

From a purely statistical perspective, one might select the operating point that maximizes the Youden index (sensitivity + specificity − 1), which identifies the threshold that optimizes overall discrimination. However, this approach does not account for disease prevalence, downstream consequences of false positives, or the clinical trade-offs inherent to screening decisions. Therefore, thresholds derived from the Youden index may not align with clinically meaningful risk thresholds, particularly in low-prevalence settings.

DCA at clinically plausible risk thresholds (e.g., 1–2%) can then reveal whether this trade-off yields a net benefit relative to “screen-all” or “screen-none” strategies. This example illustrates why AUC alone is insufficient and why explicit evaluation of operating points, calibration, and clinical utility metrics is essential for translational relevance. By integrating clinical consequences into the evaluation, DCA offers a nuanced understanding of the model’s impact on decision-making processes (Vickers and Elkin, 2006).

Besides, incorporating economic impact evaluation, such as cost-effectiveness analysis, helps to understand the financial implications of implementing the model in clinical practice. This involves comparing the costs and health outcomes of using the model against the standard of care, often using metrics such as cost per quality-adjusted life year gained (Moons et al., 2012).

Box 1 **Common pitfalls in AI-based integration of multi-layer liquid biopsy data and recommended best practices**

**Table 1:** 

Pitfall	Best practice
Sample-level splitting and batch confoundingRandomly splitting serial liquid biopsy samples can placesamples from the same patient in both training and evaluationsets. Differences between assay batches can also provideshortcuts for prediction	Split by patient and account for assay batches Keep all samples from each patient in the same partition, including across time points. Record batch membership, inspect its relationship with outcomes and partitions, and assess performance on independent batches when feasible and relevant to the intended use.
Data leakage during data preprocessing. Overly optimistic performance can arise when data preprocessing steps are conducted using the full dataset prior to data splitting.	Perform data preprocessing on the training data. All steps should be learned on the training data and then applied unchanged to the test sets.
Improper model tuning. Hyperparameter optimization performed outside the validation framework can lead to biased performance estimates, particularly in small-sample, high-dimensional settings typical of liquid biopsy studies.	Tune the models on the validation data. Ensure that no data used for final evaluation influences model tuning decisions. The test set should remain completely independent and be used only for final performance evaluation.
Insufficient reporting for reproducibility. Omitting software versions, random seeds, preprocessing settings, or hyperparameter search spaces can hinder independent replication and comparison across studies.	Document the analytical configuration. Report software/package versions, random seeds, relevant preprocessing and feature-selection settings, and the hyperparameter configurations and search spaces used during model development.
Limited interpretability and reporting. Reporting performance metrics without calibration assessment, uncertainty estimates, or a clear description of validation procedures limits clinical interpretability and transparency.	Ensure transparent reporting and interpretability. Assess calibration, apply appropriate interpretability methods, and clearly document all preprocessing and validation steps.
Key principle: All data-driven steps should be learned exclusively on the training data and applied unchanged to the test data*

*In practice, certain preprocessing steps may be challenging to repeat within each cross-validation fold due to computationally intensive procedures or methodological characteristics of particular omics data types. When such constraints arise, investigators should use a prospectively applicable alternative or report that the proposed validation does not provide an unbiased estimate of predictive performance.

## Challenges and future perspectives

This work provides methodological guidance for integrating AI-based models into the LB scenario and for addressing methodological gaps in cancer-related LB studies, including AI integration of multiomics/multimodal data. It covers the workflow from study design and preprocessing through model calibration, updating, and assessment of clinical utility. Data integration in the oncological field presents important challenges, including (i) securing high-quality and reliable data, which is essential for downstream analyses and for drawing biologically meaningful conclusions; (ii) adequate data preprocessing and exploratory analysis, often overlooked or vaguely described, which are critical steps to ensure data homogeneity and comparability across omics types, platforms, and studies; (iii) leveraging AI, implying the exploration of a variety of models, the evaluation of their performance using robust metrics, and the selection of the most appropriate approach based on the dataset characteristics and research goals; (iv) external validation and model calibration, often overlooked but essential to ensure generalizability and clinical applicability. While internal evaluation is common, external validation on independent datasets, along with proper calibration and clinically meaningful metrics, is critical for translating models into real-world settings. (v) Model interpretability is crucial, as black-box models might be unsuitable for clinical applications. We highlight tools and strategies that enhance interpretability and understanding, particularly important when integrating multiple omics layers, where insights must be biologically and clinically meaningful. (vi) Models should be regularly updated to incorporate new information, correct calibration drifts, and maintain generalizability across populations and settings, ensuring long-term robustness, adaptability, and clinical relevance. (vii) Although not covered here, data privacy and ethical concerns are critical. Handling multiomics data, particularly when linked to clinical information, raises concerns about patient privacy, data security, and informed consent. The fact that Ethical, Legal and Social Implications (ELSI) processes are not standardized and are highly fragmented (e.g., the European Union General Data Protection Regulation (GDPR) is differently interpreted by different member states) leads to significant delays in the project’s start, blocking the integration of data from larger research networks. Stringent harmonization of approval processes is, therefore, urgently needed. Aligning with the FAIR principles (Wilkinson et al., 2016) is also essential for promoting transparency, responsible reuse, and scientific integrity while protecting participant confidentiality. Moreover, AI tools in healthcare should adhere to the FUTURE principles—Fair, Universal, Traceable, Usable, Robust, and Explainable (Lekadir et al., 2025). This guideline provides strategies to ensure these principles are upheld in LB integration studies.

In recent years, LLMs have demonstrated remarkable capabilities not only for data understanding and classification but also for generation. This has given rise to the field of generative AI (GenAI), in which various models are used to generate new data within or across different modalities. Beyond natural language, GenAI now enables the creation of images, audio, and even complex biological or molecular data. In multiomics LB research, these generative capabilities could enable data augmentation, imputation of missing layers or features, synthetic data generation, and even foundation model development, opening new possibilities for LB multiomics analysis. An early step in this direction is Orion (Karimzadeh et al., 2024), a variational autoencoder applied to circulating non-coding RNAs that leverages generative sampling in the latent space to augment training representations and improve cancer classification. This demonstrates how generative modeling concepts can enhance multiomics analysis even before full-scale GenAI is applied.

## Conclusion

While still an emerging field, an increasing number of studies are integrating data from multiple layers of LB omics to address critical questions in oncology. This methods article bridges LB and AI methodologies, offering guidance to ensure studies are well designed, data are appropriately analyzed, and conclusions are biologically relevant and clinically meaningful.

As a concise set of practical recommendations, researchers are encouraged to follow the steps outlined below:

1. Clearly define the study objective and clinical context, including the intended application (e.g., early detection, prognosis, treatment response) and the target population.
2. Understand the biological and statistical properties of the data, including feature types, distributions, sparsity, and potential sources of technical variability.
3. Adopt an appropriate data-splitting and validation strategy, choosing between train-test splits, cross-validation, or nested cross-validation depending on sample size and modeling complexity.
4. Ensure that, when possible, preprocessing steps are performed on training data, including outlier handling, missing value imputation, batch-effect correction, normalization, dimensionality reduction, and feature selection, and then applied to validation or test sets.
5. Select an integration strategy and modeling approach aligned with the study objective, considering early, intermediate, or late fusion and appropriate model classes (e.g., linear models, tree-based methods, DL).
6. Report performance metrics that are appropriate for the task and data characteristics, including discrimination and calibration, and interpret them in the context of class imbalance and clinical relevance.
7. Incorporate model interpretability analyses, using appropriate global and/or local explainability methods to assess feature contributions, support biological plausibility, and facilitate clinical trust.
8. Whenever possible, perform external validation and model updating, and assess clinical utility, as these steps are critical for evaluating generalizability.

We believe that adherence to these principles will improve reproducibility, robustness, and translational relevance of AI-based integrative LB studies.

## Data Availability

The original contributions presented in the study are included in the article/Supplementary material, further inquiries can be directed to the corresponding author.

## Glossary

AUC: area under the curve
fSVA: frozen surrogate variable analysis
MAR: missing at random
MCAR: missing completely at random
MCED: multi-cancer early detection
PR: precision-recall
SMOTE: Synthetic Minority Over-sampling Technique
ROC: receiver operating characteristic
PPV: positive predictive value
AI: Artificial Intelligence
CCA: canonical correlation analysis
cfDNA: cell-free DNA
cfRNA: cell-free RNA
CTC: circulating tumor cell
ctDNA: circulating tumor DNA
ctRNA: circulating tumor RNA
DCA: decision curve analysis
DL: Deep Learning
EVs: extracellular vesicles
KNN: k-nearest neighbors
LASSO: least absolute shrinkage and selection operator
LB: liquid biopsy
LDA: linear discriminant analysis
LLM: large language model
LR: linear regression
MICE: multiple imputation by chained equations
miRNA: microRNA
ML: Machine Learning
MRD: minimal residual disease
NB: net benefit
O/E ratio: observed-to-expected ratio;
RF: random forest
TEP: tumor-educated platelet
TOO: tissue of origin
XGBoost: extreme gradient boosting
