# Supplementary material for: Learning from drops: AI-guided integration of liquid biopsy features in cancer studies
Source: Front Artif Intell. 2026 Oct 23;9:1937336. doi: 10.3389/frai.2026.1937336 (PMC13739799; doi:10.3389/frai.2026.1937336)
Supplement: Supplementary file 1 [file Supplementary_file_1.pdf]

Learning from Drops: AI-Guided Integration of Liquid Biopsy Features in Cancer Studies

Andueza et al. **Supplementary Material**

Table S1. Characteristics of the articles identified in the literature search.

Table S2. Correspondence between objectives and study designs.

Table S3. Liquid biopsy feature characteristics.

Table S4. Summary of batch-effect correction methods: characteristics, advantages, and limitations for application in liquid biopsy.

Table S5. Summary of model selection metrics: purpose, equation, advantages, and disadvantages.

Table S6. Summary of model-agnostic methods to interpret Machine Learning models.

Table S1. Characteristics of the articles identified in the literature search.

Paper	Objective	Cancer type	Study design	Covariates	Sample size/ Population	Body fluid	Liquid biopsy features	Integration methods	Model evaluation metric	Validation strategy
**(Chabon et al., 2020)**	Early detection	Lung cancer	Retrospective case-control study	Gender, age	254 patients and controls for model development and internal validation	Blood	cfDNA: mutations, CNV, methylation	Ensemble classifier using 5NN, 3NN, naive Bayes, LR, and DT	AUC, accuracy	External validation in an independent prospective cohort (temporal)
**(Gerratana et al., 2021)**	Metastatic sites identification	Breast cancer	Retrospective cohort study	Cancer stage, subtypes	88 patients with metastatic breast cancer	Blood	CTCs and ctDNA: SNVs, INDELs, gene fusions/rearrangements, CNVs	Multivariate logistic regression, RF	Accuracy, mean per-class error	Internal validation: train-test split
**(Li et al., 2021)**	Early detection	Liver cancer	Preclinical study	Tumor size	16 HCC patients, 8 HBV carriers and 32 HC	Blood	cfDNA: methylation and sequencing	DL model	Accuracy and robustness	Internal validation: train-test split
**(Ma et al., 2021)**	Early detection	Colorectal cancer	Retrospective case-control study	Gender, age	390 CRC patients and 231 HC	Blood	cfDNA: fragmentomics, end motifs, CNV	Ensemble stacked model (using 5 ML models: GLM, GBM, RF, DL, XGBoost)	ROC curve, AUC, sensitivity, specificity	Internal validation: train-test split
**(Hallermayr et al., 2022)**	Early detection and treatment response	Colorectal cancer	Retrospective case-control study	Cancer stage	50 CRC patients and 61 HC	Blood	ctDNA: fragmentomics and CNV	ML classifier	ROC curve, AUC, sensitivity, specificity	Internal validation: train-test split
**(Nakamura et al., 2022)**	Early detection	Pancreatic cancer	Retrospective cohort study	Cancer stage	168 PDAC patients and 124 HC	Blood	Cell-free circulating miRNA, exosomal miRNA and CA19-9	ML algorithms and quantitative real-time PCR assays	AUC	Internal validation: train-test split
**(Wang et al., 2022)**	Early detection and TOO classification	Lung cancer	Prospective case-control study	Gender, age, nodule size, smoking status	958 treatment naïve LUAD patients and 1318 HC	Blood	SMFs and CEA	ML models and DL	AUC, sensitivity, specificity	Internal validation: train-test split
**(Eslami-S et al., 2023)**	Prognosis prediction	Lung cancer	Prospective cohort study	Treatment, tumor histological type	54 NSCLC patients	Blood	CTCs: presence/absence and EVs: PD-L1	Multivariate Cox model	NA	No validation

**(Genco et al., 2023)**	Early detection	Pancreatic cancer	Preclinical study	NA	47 patients	Cyst fluid and blood	cfDNA: mutations and proteins	KNN algorithm	Sensitivity, specificity	Internal validation: train-test split
**(Haan et al., 2023)**	Early detection	Pancreatic cancer	Prospective case-control study	Age, gender, smoking status, stage, BMI, family history, diabetes	4408 individuals (non-cancer, pancreatic cancer and precursor lesions)	Blood	cfDNA: methylation, fragmentation	Elastic Net	AUC, sensitivity	Internal validation: train-test split. Additional internal validation in other diseases from the same cohort
**(Halner et al., 2023)**	MCED and early detection of lung cancer	Lung,breast, colorectal, oesophageal, liver, ovarian, pancreatic, gastric	Retrospective case-control study	Age, sex, ethnicity	MCED: 1005 cancer patients, 812 controls. Lung cancer: 61 lung cancer, 81 HC	Blood	Protein concentrations, cfDNA: DNA mutation 'omega' score	Data augmentation (KDE-based), feature selection (RF variable importance), multiple classifiers (RF, SVM, MLP, LR), classifier/hyperparameter optimization: Monte Carlo CV	ROC curve, AUC, sensitivity, specificity	Internal validation: hold-out test set (20%). Second external dataset to assess generalizability
**(Kwon et al., 2023)**	Early detection	Lung cancer	Retrospective case-control study	NA	92 lung cancer patients and 80 HC	Blood	cfDNA: percentage, CNV	Ensemble classifier	AUC	Internal validation: nested CV
**(Nguyen et al., 2023)**	Early detection and TOO classification	Multi-cancer	Retrospective case-control study	Gender, age, cancer stage, tumor size	738 non-metastatic patients (multi-cancer) and 1550 HC	Blood	cfDNA: methylation, CNA, fragments, end motifs	Ensemble stacking model using LR, and GCNN	AUC	Internal validation: train-test split
**(Pham et al., 2023)**	Early detection and TOO classification	Breast cancer	Retrospective case-control study	NA	239 breast cancer patients and 278 HC	Blood	cfDNA: methylation, CNA, end motifs	XGBoost	ROC curve, AUC	Internal validation: train-test split
**(Wong et al., 2023)**	Prognosis prediction	Liver cancer	Retrospective case-control study	Clinicopathologic variables (not detailed)	158 HCC patients and 33 HC	Blood	miRNA and proteins	RF	AUC, sensitivity, specificity	Internal validation: train-test split
**(Chen et al., 2025)**	Treatment response	Gastric cancer	Prospective longitudinal cohort study	Age, sex, smoking, Histology, Stage, Treatment	Training: 345 patients. External validation: 148	Blood	CTC images and circulating proteins	Deep-learning-based dynamic-aware model	ROC curve, AUC	Internal validation: train-test-split

**(Eledkawy et al., 2024)**	Early detection and TOO classification	Multi-cancer	Retrospective case-control study	Gender, age, race, histopathology	1005 patients (multi-cancer) and 812 HC	Blood	cfDNA/ctDNA: mutations and protein concentration	XGBoost, LGMB	AUC, accuracy	(Partial) External validation with a publicly available dataset (geographical)
**(van Eijck et al., 2024)**	Treatment response	Pancreatic cancer	Prospective cohort study	Age, gender and disease stage	58 PDAC patients	Blood	Plasma RNA and proteins	Stepwise logistic regression and BGLR	AUC, sensitivity, specificity	Internal validation: train-test split
**(Zhao et al., 2024)**	Early detection	Pancreatic cancer	Retrospective cohort study	NA	262 PDAC patients and 216 HC	Blood	cfDNA: methylation and CA19-9	RF	ROC curve, AUC	Internal validation: train-test split
**(Abraham et al., 2025)**	MCED, Genomic Probability Score (GPS) for diagnostic pathways, MRD and recurrence monitoring	Multi-cancer (solid tumors)	Observational, retrospective validation using multiple independent cohorts	Clinical stage, age, tumor fraction, survival outcomes, TOO, CHIP subtraction results, demographics	MCED training: 1,013 patients. Validation: 2,675 GPS training: 660 stage I/II + 506 normals (5-fold CV) MRD training: 3,439. Validation 86 (MRD) and 101 (monitoring)	Blood	Tissue: CNVs/ aneuploidy, SNVs/INDELs, fusions. LB: fragment length/dynamics, fragment diversity, nucleosome and TF footprint, end-motif, transcriptomics	XGBoost across nine feature pillars	Sensitivity, specificity, accuracy	External validation in multiple independent cohorts (geographical) for the different objectives
**(Peng et al., 2025)**	Early detection	Renall cell carcinoma (RCC)	Retrospective case-control study	NA	Train: 280 participants (142 RCC, 138 HC). Validation: 161 (81 RCC, 81 HC). External validation: 230 (90 RCC, 140 HC)	Blood	ctDNA features: CNV, fragment size distribution and nucleosome footprint	Stacked ensemble model of individual base models (GLM, RF, NN and XGBoost)	ROC curve, AUC, sensitivity, specificity	External validation in an independent cohort (geographical)
**(Tokareva et al., 2025)**	Early detection	Ovarian cancer	Retrospective case-control study	NA	229 participants (103 serous ovarian cancer, 107 benign cases, 19 HC)	Blood	Lipidomics and metabolomics	CNN model based on Mann-Whitney-selected features, XGBoost + SVM-RFE	ROC curve, AUC, accuracy, sensitivity, specificity	Internal validation
**(Van et al., 2025)**	Early detection	Breast cancer	Retrospective case-control study (training) and prospective	NA	Train: 515 female participants (273 breast cancer, 108 BBD, 134 HC). External validation:	Blood	cfDNA: mutations, CNVs, methylation	Bootstrapping (model stability assessment). SelektKBest (feature selection). Five machine learning algorithms:	ROC curve, AUC	External validation in an independent prospective cohort (temporal)

			cohort (validation)		119 females (76 breast cancer, 43 BBD)			SVM, DT, RF and XGBoost		
**(Wang et al., 2025a)**	Early detection	Oesophageal cancer	Retrospective case-control study	NA	35 oesophageal cancer patients, 110 HC	Blood	<u>cfDNA</u>: methylation, fragmentomics	LASSO, RF	ROC curve, AUC, sensitivity, specificity, MCC, F1-score, balanced accuracy	Internal validation: train-test split
**(Wang et al., 2025b)**	Prognosis prediction	Non-small cell lung cancer	Retrospective cohort study	Age, sex, smoking, histology, stage, treatment	Training: 345 patients. External validation: 148	Blood	VAF, <u>ctDNA</u> abundance, MRD, ctDNA mutations	NN, DT, random undersampling boosting, naïve Bayes, SVM and KNN	ROC curve, AUC, R1-score, accuracy, precision and recall	External validation in an independent prospective cohort (geographical)

AUC, Area Under the Curve; BBD, benign breast disease; BGLR, Bayesian Generalized Linear Regression; BMI, body mass index; CA19-9, carbohydrate antigen 19-9; CEA, carcinoembryonic antigen; cfDNA, cell-free DNA; CNVs, copy number variations; CRC, colorectal cancer; CTCs, circulating tumor cells; ctDNA, circulating tumor DNA; DL, Deep Learning; DT, decision tree; EVs, extracellular vesicles; GBM, Gradient Boosting Machine; GCNN, graph convolutional neural network; GLM, generalized linear model; GPS, genomic probability score; HBV, hepatitis B virus; HC, healthy controls; HCC, hepatocellular carcinoma; INDELs, insertions/deletions; KDE, kernel density estimation; KNN, k-nearest neighbors; LASSO, least absolute shrinkage and selection operator; LGBM: light gradient boosting machine; LR, logistic regression; LUAD, lung adenocarcinoma; MCC, Matthews Correlation Coefficient; MCED, multi-cancer early detection; miRNA, micro-RNA; ML, Machine Learning; MLP, multi-layer perceptron; MRD, minimal residual disease; NN, neural network; NSCLC, non-small cell lung cancer; PDAC, pancreatic ductal adenocarcinoma; RF, random forest; RCC; renal cell carcinoma; RFE, recursive feature elimination; ROC, Receiver Operating Characteristic; SMFs, serum metabolic fingerprints; SNV, single nucleotide variants; SVM, support vector machine; TF, transcription factor; TOO, tissue of origin; VAF, variant allele frequency; XGBoost, extreme gradient boosting.

Table S2: Study designs for different LB study objectives: proposed designs, advantages, and limitations.

Objective		Proposed study design(s)	Pros	Cons	Examples from the selected papers (Table 1)
Screening	Discovery	Case-control	Utilizes existing data. Fast and efficient	No information on temporality. Potential for missing or incomplete data; prone to confounding, bias and batch-effect	
	Validation	Prospective population cohort	Accounts for temporality	Less efficient and time-consuming	
		Nested case-control	Accounts for temporality. More efficient than a prospective cohort	Difficult to obtain if the disease has low prevalence	
(Early) Detection of a symptomatic disease	Discovery	Case-control	Utilizes existing data. Fast and efficient. Enables biomarker discovery	Does not reflect the population at risk (healthy controls are often used)	(Chabon et al., 2020)<br>(Ma et al., 2021)<br>(Hallermayr et al., 2022)<br>(Kwon et al., 2023)<br>(Wong et al., 2023)
	Validation	Case series (in a population with suspicion of disease)	Can help validate the biomarker under realistic patient conditions	Requires a prolonged case accrual period	
Detection of tumor origin	Discovery/validation	Case series (in patients with diverse cancer types)	Can help identify and validate biomarkers that identify the tumor origin	Requires a prolonged case accrual period.	(Nguyen et al., 2023)<br>(Pham et al., 2023)<br>(Eledkawy et al., 2024)
Prognosis	Discovery/validation	Case series (in patients with cancer)	Can identify prognostic factors and establish temporality	May require extended follow-up, increasing the risk of loss to follow-up	
Treatment response	Discovery	Case series	Enables exploration of treatment response in real-world settings	Prone to bias; treatment details may be inconsistent or incomplete	
	Validation	Randomised clinical trials	Gold standard for assessing treatment efficacy; minimizes confounding	Costly and resource-intensive; may not always be feasible due to logistical, ethical or recruitment constraints	

MRD detection	Discovery	Case series	Enables exploration of MRD in real-world clinical settings; useful for early biomarker identification	Prone to selection bias and limited ability to assess prognostic performance or clinical utility
	Validation	Randomised clinical trials	Allows comparison of MRD-guided interventions versus standard-of-care to evaluate their efficacy.	Costly and resource-intensive; may face ethical and logistical challenges; not always feasible in rare populations
Uncovering resistance mechanisms	Discovery/validation	Case series (in patients with cancer and under treatment)	Enables exploration of resistance mechanisms in real-world settings	Prone to bias; data might be inconsistent or incomplete
Therapeutic targets identification	Discovery	Case series (in patients with cancer)	Can help identify therapeutic targets for posterior validation	More time-consuming to conduct
	Validation	Randomised clinical trials	Gold standard for assessing treatment efficacy; minimizes confounding	Costly and time-intensive; ethical considerations if withholding therapy from control groups

Table S3: Liquid Biopsy features characteristics.

	Feature	Type of variable	Measurement Range	Data Quantity	Distribution	Reference
QUANTIFICATION	**Concentration Cell/Particle Count Presence/Absence**	Quantitative (cell count), presence/absence, concentration	0 to several hundred cells per mL	Low (a few to a few hundred cells)	Skewed with potential outliers	(Lim et al., 2019)
PROFILING	**Point Mutations**	Mutational frequency, VAF	Variant allele frequency (VAF): 0.1% to >50%	Tens to hundreds of mutations	Continuous, variable range	(Newman et al., 2016)
	**Copy Number Variations (CNVs)**	Copy number changes	Log_2 ratios or absolute copy numbers	Dozens to hundreds of CNVs	Continuous, variable range	(Heitzer et al., 2015)
	**Transcriptomics**	Gene expression levels	CPM, TPM or FPKM	High (thousands of genes/transcripts)	Continuous, expression level variability	(Ignatiadis et al., 2021)
	**Proteomics** (including secretomics)	Protein expression levels	Relative abundance or concentration	High (hundreds to thousands of proteins)	Continuous, variable range	(Zhang et al., 2014)
	**Metabolomics**	Metabolite concentrations	Nanomolar to millimolar concentrations	Moderate to high (hundreds to thousands of metabolites)	Continuous, nanomolar to millimolar	(Wishart, 2016)
	**Fragmentomics**	Fragment size, end motifs, nucleosome occupancy	Fragment length < 200 bp for ctDNA, end motifs: sequence-specific	Low abundance of ctDNA, from ≥ 5–10% in late stage to ≤ 0.01–0.1% in early-stage cancers	Continuous (fragment lengths), categorical (end motifs)	(Markus et al., 2022) (Lo et al., 2021)
	**Epigenomics**	DNA methylation (M), histone modifications, nucleosome positioning (N)	M: 0 to 100% (or 0-1) N: Varies by signal (e.g., normalized coverage depth, peak scores); indirectly reflects nucleosome occupancy	M: High (hundreds or thousands of CpG sites/histone marks). Twist panel (targeted) has less than whole genome methylation/ N: Moderate to High (depends on sequencing depth)	M: Continuous, 0 to fully methylated 1 N: Continuous (coverage signal, position-based metrics like NFR scores)	(Guo et al., 2017) (Vanderstichele et al., 2022)

Bp, Basepairs; CNV, copy number variation; CPM: counts per million; ctDNA, circulating tumor DNA; FPKM, fragments per kilobase of transcript per million mapped reads; M, methylation; N, nucleosome positioning; NFR, nucleosome-free region; TPM, transcripts per million; VAF, variant allele frequency.

Table S4: Examples of batch-effect correction methods: characteristics, advantages, and limitations for application in liquid biopsy.

Method	Input data	Description	Pros	Cons	Package / Reference
**Batch mean centering**	log_2-transformed expressions	Center data within each batch. Univariate method	-Simple and computationally efficient<br>-Requires minimal assumptions	-Non-optimal for non-Gaussian data<br>-Limited correction scope; does not account for complex batch-effects	Base R
**ComBat /ComBat-seq**	ComBat: expressions of proteomics and metabolomics<br>ComBat-seq: transcriptomics	ComBat: assumes a Gaussian distribution of the data<br>ComBat-seq: negative binomial regression to model batch-effect, mapping the data to an expected batch-free distribution	-Effective for Gaussian or count-based data<br>-Widely used and well-documented	-Performance depends on data distribution assumptions<br>-Sensitive to outliers and poor if assumptions are violated	Sva<br>https://github.com/zhangyuqing/ComBat-seq<br>(Zhang et al., 2020)
**Harmony**	Originally developed for single-cell RNA-seq data, but adaptable to other omics. Starts with a low-dimensional embedding of cells (e.g., Principal Component Analysis)	Clusters cells based on initial embedding and adjusts the embedding iteratively moving cells towards a consensus position with cells from other batches that belong to the same cluster, maintaining biological signals, and removing batch-specific variation	-Maintains biological signal integrity<br>-Effective for complex multi-batch datasets	-Computationally intensive<br>-Requires appropriate low-dimensional embeddings as input	Harmony<br>https://github.com/immunogenomics/harmony<br>(Korsunsky et al., 2019)
**Surrogate variable analysis**	Various types of expression data. Suitable for high-dimensional data and unknown batch origin	Constructs surrogate variables (covariates from high-dimensional data) which are then used to adjust for unknown, unmodeled, or latent noise sources	-Handles unknown and latent batch-effects effectively<br>-Flexible for high-dimensional data	-Requires sufficient sample sizes for surrogate variable estimation<br>-May introduce additional variability if misapplied	SVA<br>https://bioconductor.org/packages/release/bioc/html/sva.html
**Remove unwanted variation**	Various types of expression data. Suitable for high-dimensional data	Identifies and removes unwanted variation by using control genes or negative control samples	-Effective for high-dimensional data<br>-Allows explicit control through reference or control genes/samples	-Relies on accurate identification of control genes/samples<br>-May struggle with unknown batch-effects	Ruv<br>https://cran.r-project.org/web/packages/ruv/index.html
**Ratio-based scaling**	Various types of expression data. Used in proteomics by comparing the relative abundance of proteins across different batches	It is obtained by subtracting log_2 transformed expression profiles of a feature by the mean of the log_2 transformed expression profile of the reference	-Simple and easy to implement<br>-Effective for adjusting relative differences in proteomics data	-May not fully correct for complex batch-effects<br>-Relies on stable and representative reference features	Base R

Table S5: Examples of model performance metrics: purpose, equation, advantages, and disadvantages.

Metric	Purpose	Equation	Pros	Cons
**Accuracy**	Classification	$\frac{TP + TN}{TP + FP + TN + FN}$	-Simple and intuitive<br>-Works well on balanced datasets	-Misleading on imbalanced datasets.<br>-Does not differentiate between FP and FN
**Error rate**	Classification	$\frac{FP + FN}{TP + FP + TN + FN}$<br>$1 - Accuracy$	-Simple and intuitive<br>-Works well on balanced datasets	-Misleading on imbalanced datasets<br>-Does not differentiate between FP and FN
**Sensitivity/ recall/TPR**	Classification	$\frac{TP}{TP + FN}$	-Emphasizes true positive detection<br>-Useful for rare-event detection	-Ignores FP<br>-Can give a false sense of performance if precision is low
**Specificity/ TNR**	Classification	$\frac{TN}{TN + FP}$	-Emphasizes true negative detection<br>-Useful when negatives are important to classify	-Ignores FN<br>-Can give a false sense of performance if sensitivity is low
**Precision/ PPV**	Classification	$\frac{TP}{TP + FP}$	-Focuses on relevance of positive predictions<br>-Useful when FP are costly	-Ignores FN<br>-Can give misleading performance if sensitivity is low<br>-Depends on disease prevalence
**NPV**	Classification	$\frac{TN}{TN + FN}$	-Focuses on relevance of negative predictions<br>-Useful when FN are costly	-Ignores FP<br>-Can give misleading performance if specificity is low<br>-Depends on disease prevalence
**FPR**	Classification	$\frac{FP}{FP + TN}$<br>$1 - Specificity$	-Helps understand false alarm rates<br>-Complements specificity	-Alone, it does not provide information about positive class detection
**FNR**	Classification	$\frac{FN}{FN + TP}$<br>$1 - Sensitivity$	-Helps understand missed detection rates<br>-Complements sensitivity	-Alone, it does not provide information about negative class detection
**F1-score**	Classification	$2 \times \frac{Precision \times Recall}{Precision + Recall}$	-Balances precision and recall<br>-Suitable for imbalanced datasets	-Doesn't differentiate between the cost of FP and FN
**ROC curve**	Classification	*Sensitivity vs. 1 - Specificity*	-Summarizes classification performance across threshold<br>-Useful for balanced datasets	-Misleading on imbalanced datasets<br>-Focuses on ranking predictions rather than absolute performance

**AUC-ROC**	Classification	$AUC = \int_0^1 TPR(FPR)d(FPR)$	-Threshold independent<br>-Provides a single number summary	-Misleading with class imbalance<br>-Treats FPR uniformly
**PR curve**	Classification	$Precision\ vs.\ Recall$	-Better for imbalanced datasets<br>-Highlights trade-off between precision and recall	-Ignores true negatives<br>-Can be harder to interpret compared to ROC
**AUC_PR**	Classification	$AUC = \int_0^1 Precision(Recall)d(Recall)$	-Very informative for highly imbalanced datasets<br>-Focuses on positive-class performance	-Harder to interpret than ROC-AUC<br>-Baseline depends on class prevalence
**Youden's Index**	Classification	$Sensitiviy + Specificity - 1$	-Combines sensitivity and specificity<br>-Helps identify the optimal threshold	-Oversimplifies by equally weighting sensitivity and specificity
**G-mean**	Classification	$G - mean = \sqrt{Sensitivity * Specificity}$	-Useful for imbalanced datasets, not dominated by the majority class<br>-Encourages classifiers that perform well on both minority and majority classes	- Does not differentiate between the cost of FP and FN
**MCC**	Classification	$\frac{(TP \times TN) - (FP \times FN)}{\sqrt{(TP + FP)(TP + FN)(TN + FP)(TN + FN)}}$	-Balanced metric considering all confusion matrix elements<br>-Works well for imbalanced datasets	-Computationally intensive<br>-Hard to interpret compared to simpler metrics like accuracy
**LR⁺**	Classification	$\frac{Sensitivity}{1 - Specificity}$	-Quantifies how much a positive result increases disease likelihood	-Requires both sensitivity and specificity<br>-May be challenging to interpret directly
**LR⁻**	Classification	$\frac{1 - Sensitivity}{Specificity}$	-Quantifies how much a negative result decreases disease likelihood	-Requires both sensitivity and specificity<br>-May be challenging to interpret directly
**DOR**	Classification	$\frac{LR^+}{LR^-}$<br>$\frac{TP \times TN}{FP \times FN}$	-Combines sensitivity and specificity into a single number	-Does not provide information about specific error types
**Micro-AUC**	Classification	AUC−ROC obtained by aggregating all class predictions and labels across classes	-Aggregates all predictions<br>-Works well for overall performance	-Dominated by majority classes, making it less informative for imbalanced datasets
**Macro-AUC**	Classification	$\frac{1}{k}\sum_{i=1}^{k} AUC_i$	-Treats all classes equally<br>-Suitable for comparing performance across classes	-Ignores class imbalance<br>-May overemphasize minority classes with poor performance

**MAE**	Regression	$\frac{1}{n}\sum_{i=1}^{n}\lvert y_i - ypred_i \rvert$	-Easy to interpret; errors are in the same unit as the original data<br>-Robust to outliers compared to MSE and RMSE<br>-Provides a straightforward average error magnitude	-Does not penalize large errors more than small errors (lacks quadratic emphasis)<br>-May not be suitable when large errors need greater attention
**MSE**	Regression	$\frac{1}{n}\sum_{i=1}^{n}(y_i - ypred_i)^2$	-Penalizes large errors more than small ones, making it useful for capturing significant deviations<br>-Differentiable, making it suitable for optimization in machine learning	-Not in the same units as the original data (squared scale)<br>-Sensitive to outliers, as large errors are squared
**RMSE**	Regression	$\sqrt{\frac{1}{n}\sum_{i=1}^{n}(y_i - ypred_i)^2}$	-In the same units as the original data, making it easier to interpret than MSE<br>-Penalizes large errors like MSE, which is useful when outliers are important	-Still sensitive to outliers due to squaring errors<br>-More computationally expensive because of the square root
**R^2**	Regression	$1 - \frac{\sum_{i=1}^{n}(y_i - ypred_i)^2}{\sum_{i=1}^{n}(y_i - \overline{y})^2}$	-Indicates how well the model explains the variance in the target variable<br>-Normalized between 0 and 1 for easy interpretation	-Can give misleading results if the model is overfitted or if the data is non-linear<br>-Not intuitive for understanding absolute error magnitudes
**Adjusted R^2**	Regression	$1 - \left(\frac{(1 - R^2)(n-1)}{n-k-1}\right)$	-Penalizes overfitting by accounting for the number of predictors<br>-Provides a more realistic measure of model performance for multiple predictors	-Can still overestimate performance if irrelevant predictors are included<br>-Slightly more complex to calculate and interpret compared to R^2
**C-index**	Time-to-event discrimination	$C = \frac{\sum_{i \neq j} I(T_i < T_j, \delta_i = 1)[I(r_i > r_j) + \frac{1}{2} I(r_i = r_j)]}{\sum_{I \neq j} I(T_i < T_j, \delta_i = 1)}$	Summarizes ranking of predicted risk while accounting for censoring	Does not assess calibration or performance at a particular clinical time horizon; estimator depends on censoring assumptions
**Time dependent AUC at t**	Time-to-event discrimination at time t	$AUC(t) = P(r_i > r_j \mid T_i^* \leq t, T_j^* > t)$	Shows discrimination at clinically relevant horizons	Depends on the chosen horizon and definition of cases and controls; requires adequate follow-up
**Observed-to-expected event risk at t**	Agreement between predicted and	$O/E(t) = \frac{1 - S_{obs}(t)}{\frac{1}{N}\sum_{i=1}^{N} p_i(t)}$	Reveals overprediction or underprediction of absolute risk	Results depend on the chosen horizon and reliable estimation of observed risk

observed event<br>risk	

AUC, area under the curve; DOR, diagnostic odds ratio; LR^+, positive diagnostic likelihood ratio; LR^-, negative diagnostic likelihood ratio; FN, false negative; FNR, false negative rate; FP, false positive; FPR, false positive rate; k, number of classes; MAE, mean absolute error; MCC, Matthews Correlation Coefficient; MSE, mean square error; n, number of samples; NPV, negative predictive value; PPV, positive predictive value; PR, precision-recall; R^2, R squared; RMSE, root mean squared error; ROC, receiver operation characteristic; TN, true negative; TNR, true negative rate; TP, true positive; TPR, true positive rate

Table S6: Examples of model-agnostic methods to interpret ML models.

	Method	Model Explanation	Pros	Cons
Global methods	Partial dependence plot	Shows the marginal effect of 1 or 2 features on the predicted outcome	-Easy to interpret<br>-Useful for assessing feature importance	-Assumes independence between features, which may not hold in practice<br>-Limited to 1-2 features
	Accumulated local effects plot	Describes how a feature affects the average prediction	-Accounts for feature dependencies<br>-Computationally efficient compared to PDP	-Still limited to feature-specific effects<br>-May miss nuanced interactions
	Feature interactions	H-Statistic. Measures the interaction between two variables or between one variable and the rest. It has meaningful interpretation and works with all kinds of interactions. Computationally expensive	-Quantifies interactions effectively<br>-Broad applicability across interaction types	-High computational cost for large datasets<br>-Difficult to interpret with complex interactions
	Functional decomposition	Deconstructs the high-dimensional function as a sum of individual effects and interaction effects	-Provides a detailed breakdown of feature contributions<br>-Captures complex interactions	-Challenging to implement<br>-Interpretation becomes harder with increasing dimensions
	Permutation feature importance	Analyzes the increase in prediction error after permuting the feature's values	-Provides clear feature importance rankings	-Sensitive to correlated features<br>-Can underestimate the importance of features involved in complex interactions
	Global surrogate models	Trains interpretable models to approximate the predictions of a black-box model	-Intuitive approach to explainability<br>- Works with any black-box model	-May lose fidelity when approximating complex models<br>-Choice of surrogate impacts interpretability
	Prototypes and criticisms	Maximum Mean Discrepancy. It finds prototypes (well-representative data instances) and criticisms (data instances not well-represented by prototypes)	-Helps identify representative and outlier instances<br>-Offers insights into data structure	-Computationally expensive for large datasets<br>-Prototype selection can be subjective

Local Methods	Individual conditional expectation	Visualizes how the prediction of a ML model changes for an individual instance as a specific feature varies	-Provides instance-level insights<br>-Highlights heterogeneous effects across instances	-Limited to one feature at a time<br>-Can be visually complex for high-dimensional data
	Local interpretable model-agnostic explanations (LIME)	Interpretable models are used to explain individual predictions made by a black-box model	-Simple, intuitive, and local interpretability<br>-Works for any model type	-Sensitive to sampling strategy<br>-May not generalize well to other instances
	Counterfactual explanations	Identifies how to minimally alter an individual instance's features to change its prediction to a desired outcome, providing insight into the model's decision boundary	-Useful for actionable insights<br>-Provides a clear sense of decision boundaries	-Computationally expensive for high-dimensional data<br>-May yield unrealistic or non-actionable suggestions
	Scoped rules (anchors)	Generates if-then rules (anchors), highlighting conditions under which a model consistently makes the same prediction	-Produces interpretable, human-readable rules<br>-Focuses on high-confidence decision regions	-Rule generation may be infeasible for highly complex models<br>-Rules may oversimplify decision logic
	Shapley values	Quantify each feature's contribution to a prediction by considering all possible feature combinations, offering a fair, cooperative game theory-based measure of feature importance	-Fair and theoretically sound (game-theoretic)<br>-Handles correlated features better than simpler importance measures	-Computationally expensive, especially for high-dimensional models
	SHAP	Combines Shapley values with local explanations, offering a fair measure of feature importance by attributing each feature's contribution to individual predictions	-Balances local and global interpretability<br>-Robust for understanding complex models	-Computational cost can be high for large datasets or models<br>-Interpretation may be challenging for non-experts

LIME, local interpretable model-agnostic explanations; ML, machine learning; PDP, partial dependence plot; SHAP, Shapley additive explanations.

References

Abraham, J., Domenyuk, V., Perdigones, N., Klimov, S., Antani, S., Yoshino, T., et al. (2025). Validation of an AI-enabled exome/transcriptome liquid biopsy platform for early detection, MRD, disease monitoring, and therapy selection for solid tumors. *Sci. Rep.* 15:21173. doi: 10.1038/s41598-025-08986-0

Chabon, J. J., Hamilton, E. G., Kurtz, D. M., Esfahani, M. S., Moding, E. J., Stehr, H., et al. (2020). Integrating genomic features for non-invasive early lung cancer detection. *Nature* 580, 245–251. doi: 10.1038/s41586-020-2140-0

Chen, Z., Zhao, J., Li, Y., Feng, X., Chen, Y., Li, Y., et al. (2025). Predicting response to patients with gastric cancer via a dynamic-aware model with longitudinal liquid biopsy data. *Gastric Cancer* 28, 886–898. doi: 10.1007/s10120-025-01628-4

Eledkawy, A., Hamza, T., and El-Metwally, S. (2024). Precision cancer classification using liquid biopsy and advanced machine learning techniques. *Sci. Rep.* 14:5841. doi: 10.1038/s41598-024-56419-1

Eslami-S, Z., Cortés-Hernández, L. E., Sinoquet, L., Gauthier, L., Vautrot, V., Cayrefourcq, L., et al. (2023). Circulating tumour cells and PD-L1-positive small extracellular vesicles: the liquid biopsy combination for prognostic information in patients with metastatic non-small cell lung cancer. *Br. J. Cancer* 130, 63–72. doi: 10.1038/s41416-023-02491-9

Genco, E., Modena, F., Sarcina, L., Björkström, K., Brunetti, C., Caironi, M., et al. (2023). A single-molecule bioelectronic portable array for early diagnosis of pancreatic cancer precursors. *Adv Mater* 35, 2304102. doi: 10.1002/adma.202304102

Gerratana, L., Davis, A. A., Polano, M., Zhang, Q., Shah, A. N., Lin, C., et al. (2021). Understanding the organ tropism of metastatic breast cancer through the combination of liquid biopsy tools. *Eur. J. Cancer* 143, 147–157. doi: 10.1016/j.ejca.2020.11.005

Guo, S., Diep, D., Plongthongkum, N., Fung, H. L., Zhang, K., and Zhang, K. (2017). Identification of methylation haplotype blocks AIDS in deconvolution of heterogeneous tissue samples and tumor tissue-of-origin mapping from plasma DNA. *Nat. Genet.* 49, 635–642. doi: 10.1038/ng.3805

Haan, D., Bergamaschi, A., Friedl, V., Guler, G. D., Ning, Y., Reggiardo, R., et al. (2023). Epigenomic blood-based early detection of pancreatic cancer employing cell-Free DNA. *Clinical Gastroenterology and Hepatology* 21, 1802-1809.e6. doi: 10.1016/j.cgh.2023.03.016

Hallermayr, A., Wohlfrom, T., Steinke-Lange, V., Benet-Pagès, A., Scharf, F., Heitzer, E., et al. (2022). Somatic copy number alteration and fragmentation analysis in circulating tumor DNA for cancer screening and treatment monitoring in colorectal cancer patients. *J. Hematol. Oncol.* 15:125. doi: 10.1186/s13045-022-01342-z

Halner, A., Hankey, L., Liang, Z., Pozzetti, F., Szulc, D., Mi, E., et al. (2023). DEcancer: Machine learning framework tailored to liquid biopsy based cancer detection and biomarker signature selection. *iScience* 26:106610. doi: 10.1016/j.isci.2023.106610

Heitzer, E., Ulz, P., and Geigl, J. B. (2015). Circulating tumor DNA as a liquid biopsy for cancer. *Clin. Chem.* 61, 112–123. doi: 10.1373/clinchem.2014.222679

Ignatiadis, M., Sledge, G. W., and Jeffrey, S. S. (2021). Liquid biopsy enters the clinic — implementation issues and future challenges. *Nat. Rev. Clin. Oncol.* 18, 297–312. doi: 10.1038/s41571-020-00457-x

Korsunsky, I., Millard, N., Fan, J., Slowikowski, K., Zhang, F., Wei, K., et al. (2019). Fast, sensitive and accurate integration of single-cell data with Harmony. *Nat. Methods* 16, 1289–1296. doi: 10.1038/s41592-019-0619-0

Kwon, H. J., Park, U. H., Goh, C. J., Park, D., Lim, Y. G., Lee, I. K., et al. (2023). Enhancing lung cancer classification through integration of liquid biopsy multi-omics data with machine learning techniques. *Cancers (Basel).* 15. doi: 10.3390/cancers15184556

Li, J., Zhang, X., Zhang, W., Wang, H., Zhong, B., Xie, Z., et al. (2021). DISMIR: Deep learning-based noninvasive cancer detection by integrating DNA sequence and methylation information of individual cell-free DNA reads. *Brief. Bioinform.* 22:bbab250. doi: 10.1093/BIB/BBAB250

Lim, S. Bin, Di Lee, W., Vasudevan, J., Lim, W. T., and Lim, C. T. (2019). Liquid biopsy: one cell at a time. *NPJ Precis. Oncol.* 3, 1–9. doi: 10.1038/s41698-019-0095-0

Lo, Y. M. D., Han, D. S. C., Jiang, P., and Chiu, R. W. K. (2021). Epigenetics, fragmentomics, and topology of cell-free DNA in liquid biopsies. *Science (1979).* 372:6538. doi: 10.1126/science.aaw3616

Ma, X., Chen, Y., Tang, W., Bao, H., Mo, S., Liu, R., et al. (2021). Multi-dimensional fragmentomic assay for ultrasensitive early detection of colorectal advanced adenoma and adenocarcinoma. *J. Hematol. Oncol.* 14:175. doi: 10.1186/s13045-021-01189-w

Markus, H., Chandrananda, D., Moore, E., Mouliere, F., Morris, J., Brenton, J. D., et al. (2022). Refined characterization of circulating tumor DNA through biological feature integration. *Sci. Rep.* 12, 1–11. doi: 10.1038/s41598-022-05606-z

Nakamura, K., Zhu, Z., Roy, S., Jun, E., Han, H., Munoz, R. M., et al. (2022). An exosome-based transcriptomic signature for noninvasive, early detection of patients with pancreatic ductal adenocarcinoma: A multicenter cohort study. *Gastroenterology* 163, 1252-1266.e2. doi: 10.1053/j.gastro.2022.06.090

Newman, A. M., Lovejoy, A. F., Klass, D. M., Kurtz, D. M., Chabon, J. J., Scherer, F., et al. (2016). Integrated digital error suppression for improved detection of circulating tumor DNA. *Nat. Biotechnol.* 34, 547–555. doi: 10.1038/nbt.3520

Nguyen, V. T. C., Nguyen, T. H., Doan, N. N. T., Pham, T. M. Q., Nguyen, G. T. H., Nguyen, T. D., et al. (2023). Multimodal analysis of methylomics and fragmentomics in plasma cell-free DNA for multi-cancer early detection and localization. eLife 12:RP89083. doi: 10.7554/elife.89083

Peng, Y. L., Yu, B., Huang, T. X., Zhou, Z. H., Zhang, H., Tang, W. X. F., et al. (2025). Early detection of renal cell carcinoma: a novel cell-free DNA fragmentomics-based liquid biopsy assay. *ESMO Open* 10:105323. doi: 10.1016/j.esmoop.2025.105323

Pham, T. M. Q., Phan, T. H., Jasmine, T. X., Tran, T. T. T., Huynh, L. A. K., Vo, T. L., et al. (2023). Multimodal analysis of genome-wide methylation, copy number aberrations, and end motif signatures enhances detection of early-stage breast cancer. *Front. Oncol.* 13:1127086. doi: 10.3389/fonc.2023.1127086

Tokareva, A., Iurova, M., Starodubtseva, N., Chagovets, V., Novoselova, A., Kukaev, E., et al. (2025). Machine learning framework for ovarian cancer diagnostics using plasma lipidomics and metabolomics. *Int. J. Mol. Sci.* 26:6630. doi: 10.3390/ijms26146630

van Eijck, C. W. F., Sabroso-Lasa, S., Strijk, G. J., Mustafa, D. A. M., Fellah, A., Koerkamp, B. G., et al. (2024). A liquid biomarker signature of inflammatory proteins accurately predicts early pancreatic cancer progression during FOLFIRINOX chemotherapy. *Neoplasia* 49:100975. doi: 10.1016/j.neo.2024.100975

Van, T. T. V., Tran, T. H., Nguyen, T. H. H., Nguyen, V. T. C., Vo, D. H., Nguyen, G. T. H., et al. (2025). Multimodal analysis of cell-free DNA enhances differentiation of early-stage breast cancer from benign lesions and healthy individuals. *BMC Biol.* 23:259. doi: 10.1186/s12915-025-02371-z

Vanderstichele, A., Busschaert, P., Landolfo, C., Olbrecht, S., Coosemans, A., Froyman, W., et al. (2022). Nucleosome footprinting in plasma cell-free DNA for the pre-surgical diagnosis of ovarian cancer. *NPJ Genom. Med.* 7, 1–9. doi: 10.1038/s41525-022-00300-5

Wang, J., Zhang, W., Li, H., Wang, Y., Qi, J., and Nie, J. (2025a). Cell-free DNA methylation and fragmentomics-based liquid biopsy for accurate esophageal cancer detection. *BMC Cancer* 25:1816. doi: 10.1186/s12885-025-15150-4

Wang, L., Zhang, M., Pan, X., Zhao, M., Huang, L., Hu, X., et al. (2022). Integrative serum metabolic fingerprints based multi-modal platforms for lung adenocarcinoma early detection and pulmonary nodule classification. *Adv Sci (Weinh)* 9:2203786. doi: 10.1002/advs.202203786

Wang, Y., Xiang, Y. B., Chen, X. W., Zhang, T., Wang, J. Y., Liu, W. Y., et al. (2025b). PRIME: an interpretable artificial intelligence model based on liquid biopsy improves prediction of progression risk in non-small cell lung cancer. *Mil. Med. Res.* 12:94. doi: 10.1186/s40779-025-00679-z

Wishart, D. S. (2016). Emerging applications of metabolomics in drug discovery and precision medicine. *Nat. Rev. Drug Discov.* 15, 473–484. doi: 10.1038/nrd.2016.32

Wong, V. C.-L., Wong, M.-I., Lee, V. H.-F., Man, K., Ng, K. T.-P., and Cheung, T. T. (2023). Prognostic MicroRNA fingerprints predict recurrence of early-stage hepatocellular carcinoma following hepatectomy. *J. Cancer* 14, 480–489. doi: 10.7150/jca.79593

Zhang, B., Wang, J., Wang, X., Zhu, J., Liu, Q., Shi, Z., et al. (2014). Proteogenomic characterization of human colon and rectal cancer. *Nature* 513, 382–387. doi: 10.1038/nature13438

Zhang, Y., Parmigiani, G., and Johnson, W. E. (2020). ComBat-seq: batch-effect adjustment for RNA-seq count data. *NAR Genom. Bioinform.* 2:lqaa078. doi: 10.1093/nargab/lqaa078

Zhao, G., Jiang, R., Shi, Y., Gao, S., Wang, D., Li, Z., et al. (2024). Circulating cell-free DNA methylation-based multi-omics analysis allows early diagnosis of pancreatic ductal adenocarcinoma. *Mol. Oncol.* 18:2801. doi: 10.1002/1878-0261.13643
